# An Injectable Kartogenin-Incorporated Hydrogel Supports Mesenchymal Stem Cells for Cartilage Tissue Engineering

**DOI:** 10.3390/bioengineering12050434

**Published:** 2025-04-22

**Authors:** Chongquan Huang, Guoqing Zhong, Jin Xiao, Xiaolan Wang, Weijuan Huang, Lingyun Chen, Yu Zhang, Shi Cheng

**Affiliations:** 1 Department of Orthopedics, Guangdong Provincial People’s Hospital (Guangdong Academy of Medical Sciences), Southern Medical University, Guangzhou 510080, China; hcq4400@163.com (C.H.);; 2 The Second School of Clinical Medicine, Southern Medical University, Guangzhou 510515, China; 3 Guangdong Engineering Technology Research Center of Functional Repair of Bone Defects and Biomaterials, Guangzhou 510080, China; 4 Department of Agricultural, Food & Nutritional Science, University of Alberta, Edmonton, AB T6G 2P5, Canada; 5 College of Food Science, South China Agricultural University, Guangzhou 510642, China

**Keywords:** cartilage tissue engineering, injectable hydrogel, chitosan, cellulose nanocrystal, tissue regeneration

## Abstract

Background: Cartilage defects and injuries often lead to osteoarthritis, posing significant challenges for cartilage repair. Traditional treatments have limited efficacy, necessitating innovative therapeutic strategies. This study aimed to develop an injectable hydrogel-based tissue engineering construct to enhance cartilage regeneration by combining mesenchymal stem cells (MSCs) and the small molecule drug kartogenin (KGN). Methods: An injectable hydrogel was synthesized by crosslinking carboxymethyl chitosan (CMC) with aldehyde-modified cellulose nanocrystals (DACNCs). KGN was incorporated into the hydrogel during crosslinking to achieve sustained drug release. Three hydrogels with varying CMC/DACNC molar ratios (MR = 0.11, 0.22, and 0.33) were developed and characterized for their structural, mechanical, and biocompatible properties. The hydrogel with the optimal ratio (MR = 0.33) was further evaluated for its ability to support MSC viability and differentiation in vitro. Additionally, signaling pathways (TGF-β, FOXO, and PI3K-AKT) were investigated to elucidate the underlying mechanisms. In vivo efficacy was assessed using a rabbit femoral trochlear cartilage defect model. Results: The hydrogel with a higher CMC/DACNC molar ratio (MR = 0.33) exhibited increased compressive modulus, a reduced swelling rate, and superior biocompatibility, effectively promoting MSC differentiation in vitro. Signaling pathway analysis revealed activation of the TGF-β, FOXO, and PI3K-AKT pathways, suggesting enhanced chondrogenic potential. In vivo experiments demonstrated that the KGN-MSC-encapsulated hydrogel significantly improved cartilage repair. Conclusions: The injectable CMC/DACNC hydrogel, combined with KGN and MSCs, synergistically enhanced cartilage regeneration both in vitro and in vivo. This study highlights the potential of this hydrogel as a promising scaffold for cartilage tissue engineering, offering a novel therapeutic approach for cartilage defects and injuries.

## 1. Introduction

Cartilage defects stemming from trauma, osteoarthritis, or aging present substantial challenges on a societal and economic scale globally [1,2]. Despite clinical efforts, regenerating cartilage remains a formidable task due to its limited inherent healing capacity [3,4]. Current clinical approaches like microfracture surgery and grafting, while common, have drawbacks such as the formation of mechanically inferior fibrocartilage post-microfracture and limitations related to patient age and graft availability [5]. Advancements in tissue engineering have introduced promising strategies for cartilage repair, focusing on mesenchymal stem cells (MSCs), bioactive factors, and various scaffold materials. To date, many kinds of innovative biomaterials have been developed in the field of cartilage repair and reconstruction including 3D-printed scaffolds [6], hydrogels [7,8], or nanoparticles [9,10]. Hydrogels, in particular, are highly regarded for their ability to serve as carriers for stem cells and growth factors, thanks to their hydrophilic nature and capacity for swelling and hydration [11,12,13]. Moreover, their structural resemblance to the extracellular matrix is conducive to cell viability, proliferation, and differentiation, offering potential through physical and chemical cues.

Significant advancements have been made in the application of hydrogels for cartilage regeneration. Various natural and synthetic polymers, including collagen, hyaluronic acid, chitosan, and poly(lactic-co-glycolic acid), have been explored for developing hydrogel scaffolds for cartilaginous tissue engineering. Chitosan, a natural polysaccharide derived from renewable resources, shares a molecular structure similar to glycosaminoglycans and exhibits desirable biocompatibility and chondro-conducive properties [14]. These attributes make chitosan a promising base material for hydrogels to regenerate cartilaginous tissue. However, its poor solubility in neutral water significantly limits its application. Moreover, crosslinking agents like β-glycerophosphate can increase toxicity and compromise the injectability of chitosan-based hydrogels. To address these limitations, various chitosan derivatives have been developed. Carboxymethyl chitosan (CMC), one such derivative, offers high solubility in physiological conditions and contains abundant amine groups that readily react with aldehyde groups to form hydrogels [15]. This makes CMC a more suitable base material for cartilage regeneration compared with chitosan. Nonetheless, an ideal crosslinking agent is essential to facilitate non-toxic and highly tunable hydrogel formation. Cellulose nanocrystal (CNC) is an emerging biobased nanomaterial with good biocompatibility and dispersibility. Aldehyde-modified CNCs (DACNCs) retain outstanding mechanical strength and stiffness while offering ample aldehyde groups for crosslinking. In our previous research, we successfully fabricated an injectable, self-healing hydrogel by combining DACNCs and CMC through Schiff-base linkages [16]. This hydrogel demonstrated good biocompatibility, swelling capacity, and injectability, making it an effective biomaterial carrier for delivering human bone marrow-derived mesenchymal stem cells (hBMSCs) to aid in cartilage regeneration. The hydrogel’s injectability and in situ polymerization via Schiff-base linkages are particularly advantageous for minimally invasive treatments as they prevent iatrogenic damage to normal tissue and eliminate the need for secondary procedures such as ultraviolet radiation or increased temperature. However, varying the ratio of CMC to DACNC alters the hydrogel’s mechanical properties, which are crucial for influencing the proliferation and chondrogenic differentiation of stem cells [17,18]. Therefore, determining the optimal mechanical characteristics of CMC/DACNC hydrogels for cartilage regeneration is a critical and urgent objective.

**Figure 1:**
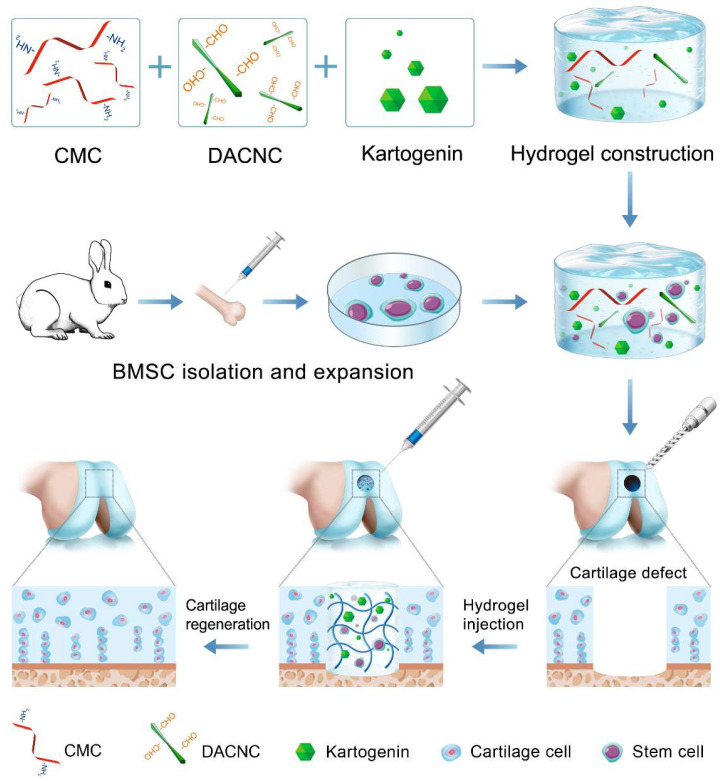
This study synthesized an injectable self-healing hydrogel via dynamic Schiff-base crosslinking between CMC and DACNC and incorporated the small molecule drug KGN to enhance the chondrogenic differentiation of stem cells. This hydrogel exhibits good biocompatibility, tunable mechanical properties, and controlled drug release capabilities. Through comprehensive in vitro and in vivo experiments, we demonstrated its superiority in promoting stem cell proliferation, cartilage matrix secretion, and cartilage repair quality.

Bioactive factors play a crucial role in cartilage tissue engineering. Bioactive proteins, such as transforming growth factor β (TGF-β), have been extensively reported as effective therapeutic agents for enhancing MSC-based cartilage regeneration [19,20]. However, their application in vivo is hampered by their short half-life and susceptibility to denaturation. Recently, small molecule drugs have garnered significant attention for cartilage regeneration due to their stable structure and effectiveness. Kartogenin (KGN), a prominent nonprotein small molecule, has been identified as a potent agent for chondrogenic differentiation [21,22]. KGN exhibits significant chondrogenesis-inducing effects without toxicity, and its stable chemical properties allow for long-term storage and easy encapsulation into hydrogels, thereby overcoming the limitations associated with protein factors. Considering its superior role in cartilage regeneration, KGN was loaded in CMC/DACNC hydrogels to provide a suitable environment for stem cell proliferation and differentiation (Figure 1). Additionally, transcriptome sequencing was employed to investigate the mechanism through which materials influence cartilage differentiation. We hypothesized that the composite injectable hydrogels loaded with KGN could promote the chondrogenic differentiation of stem cells, thus promoting cartilage regeneration.

## 2. Materials and Methods

### 2.1. Materials

All materials and preparation methods used in this study were identical to our previous study [16]. Chitosan (viscosity-average molecular weight: 12.4 × 10^5^, degree of deacetylation: 72%) was purchased from Sigma-Aldrich (St. Louis, MO, USA). Spruce cellulose (bleached kraft pulp, Mw = 4.10 × 10^5^ g/mol) with an α-cellulose content of 87.3% was provided by Alberta Pacific Forest Industries Inc. (Boyle, AB, Canada). The content of the primary amino group in CMC was 0.56 mmol/g CMC and the degree of carboxymethyl substitution (DS) of CMC was 0.31, as described in our previous study [16]. DACNC was prepared with sulfuric acid hydrolysis followed by periodate oxidation of wood cellulose, and its length was 114 ± 29 nm. KGN was supplied by MCE Reagent (Shanghai, China).

Sprague Dawley (SD) male rats weighing 300–350 g and 8-week-old male New Zealand rabbits were purchased from the Medical Experimental Animal Center of Guangdong Province. The animal experiments were conducted under the authorization of the Guidelines for the Care and Use of Laboratory Animals and the Animal Ethics Committee of Guangdong Provincial People’s Hospital (Ethical Approval Protocol No. KY2023-867-01, issued on 20 November 2023), and in accordance with the National Research Council’s Guide for the Care and Use of Laboratory Animals.

### 2.2. Synthesis of CMC/DACNC Hydrogels

The fabrication of CMC, DACNC, and the synthesis of CMC/DACNC hydrogels were described in our previous study [16]. Briefly, a 4 wt.% CMC solution was prepared by dissolving CMC powder in phosphate buffer solution (PBS) and stirring overnight at room temperature. Similarly, a 4 wt.% DACNC solution was prepared by dispersing 400 mg of DACNC powder in 9.6 g of PBS and stirring in an 80 °C water bath for 4 h. The CMC/DACNC hydrogels were synthesized by homogeneously mixing the CMC solution with the DACNC solution at room temperature, using different molar ratios (MR = n (amines from CMC)/n (aldehydes from DACNC)) of 3, 1, and 0.33. Gelation was confirmed using the vial tilting method. The hydrogels were categorized based on their stiffness: high stiffness (MR = 0.33), middle stiffness (MR = 1), and low stiffness (MR = 3).

### 2.3. Material Characterization

#### 2.3.1. Scanning Electron Microscope (SEM) Observation

The morphologies of CMC/DACNC hydrogels were characterized under a scanning electron microscope (SEM, Morgagni 268, Philips-FEI, Hillsboro, OR, USA). Fabricated hydrogels were cut into small circular disks and then freeze-dried and sputter-coated with gold. These were then observed under a scanning electron microscope. Pore sizes of the hydrogels were calculated using ImageJ software (NIH, Bethesda, MD, USA; version 1.54g).

#### 2.3.2. Mechanical Properties Assay

Mechanical properties of the high, middle, and low stiffness hydrogels that were about 20 mm in diameter and 10 mm in height were determined using an Instron 5967 universal testing instrument (Instron Corp, Norwood, MA, USA) equipped with a 50 N load cell. All gel samples were compressed to 50% of their original height at room temperature and with a constant crosshead speed of 1 mm/min. Compressive modulus refers to the slope of the initial linear region (elastic range, ε = 0–10%) of the stress–strain curve.

#### 2.3.3. Swelling Ratio and Injectable Analysis

To study the swelling kinetics of the hydrogels, hydrogels were cut into discs 20 mm in diameter and 6 mm in height and immersed in 10 mL PBS at 37 °C. After default time intervals, samples were removed from PBS and the excess water on the surfaces was gently blotted with a piece of paper towel. The weight of the samples was measured and compared with the initial weight.

Hydrogel samples were prepared by loading them into 5 mL syringes equipped with 20-gauge needles (inner diameter 0.6 mm). The extrusion process was conducted manually at a controlled speed of ≈1 mL/min, with the flow rate calibrated through volume displacement measurements. Successful injection was characterized by continuous hydrogel flow without observable phase separation or needle clogging during the extrusion process.

### 2.4. Cell Culture

Rabbit bone mesenchymal stem cells (RBMSCs) were freshly isolated from the bone marrow of neonatal rabbits, following protocols from previous studies [23,24]. All animal procedures complied with the regulations and guidelines of the Ethics Committee of Guangdong Provincial People’s Hospital and adhered to the Institutional Animal Care and Use Committee (IACUC) guidelines. In summary, bone marrow was aseptically harvested from the femur condyle and tibia of anesthetized four-week-old male New Zealand rabbits. The bone marrow was mixed with PBS at a 1:1 ratio and allowed to stand for 15 min. Following centrifugation at 1000 rpm for 5 min, the supernatant was discarded, and the pellet was resuspended in PBS. An equal volume of Ficoll-PaqueH (Sigma, St. Louis, MO, USA) stem cell isolation liquid was then added to the centrifuge tube. After 20 min of centrifugation at 2000 rpm, mononuclear cells were collected from the interphase (cloud-like cell layer) and cultured in a 25 cm^2^ flask with a basal RBMSC culture medium (Cyagen, Guangzhou, China), supplemented with 10% fetal bovine serum (FBS, Gibco BRL, Grand Island, NY, USA) and 1% penicillin-streptomycin (Hyclone, Logan, UT, USA) in a humidified incubator at 37 °C and 5% CO_2_. After 12 h, non-adherent cells were removed and the medium was replaced with a fresh complete medium. The culture medium was changed every two days. When the cells reached 70–80% confluence, they were harvested using 0.25% trypsin-EDTA (Gibco, Grand Island, NY, USA) and subcultured at a 1:3 ratio. Cells at passage 2 were used for subsequent experiments.

Primary cells were identified as RBMSCs through morphological observation and trilineage differentiation capacity. Phase contrast microscopy revealed a characteristic spindle-shaped morphology (Supplementary Figure S12a). Multipotency was validated through adipogenic (Oil Red O staining), osteogenic (Alizarin red staining), and chondrogenic (Alcian blue staining) induction, with the results shown in Supplementary Figure S12b–d.

### 2.5. Cell Viability and Proliferation Analysis

To encapsulate the cells into hydrogels, the CMC and DACNC powders were first dissolved in PBS, and then the DACNC solution was used to suspend the cells. The CMC solution and the DACNC solution containing cells were injected into a 24-well plate at different molar ratios (3, 1, 0.33) and mixed with a pipette to form the hydrogel (5 × 10^5^ cells/mL hydrogel). The plate was cultured in an incubator at 37 °C with 5% CO_2_. After 1, 3, 5, and 7 days of culture, a CCK-8 assay was performed to assess the cell proliferation. Briefly, the medium was replaced with 110 μL of prepared CCK-8 working solution and incubated for 4 h. The reaction mixture was then transferred into a 96-well plate and measured at 450 nm with a microplate reader.

On days 3 and 7, cell viability within the hydrogels was observed using Calcein-AM/Propidium Iodide (Calcein-AM/PI, Invitrogen, Carlsbad, CA, USA) staining. For plate culture, hydrogels were first dissolved in glycine aqueous solution (100 mg/mL) at 37 °C for 1 h to release the entrapped cells. The cells were then cultured in a plate and stained with Calcein-AM/PI. For direct observation, hydrogels were incubated with staining solution for 20 min at 37 °C and 5% CO_2_. Samples were washed with PBS twice and visualized using a CLSM 710 Meta confocal laser scanning microscope (Carl Zeiss, Jena, Germany). Images and 3D confocal stacks were processed with ZEN 2012 SP5 software (Carl Zeiss MicroImaging GmbH, Jena, Germany, Version 14.0.0.0). The percentage of live cells was calculated by the ratio of live cell number to total cell number from three random views.

### 2.6. In Vitro KGN-Induced Chondrogenic Differentiation

#### 2.6.1. In Vitro Drug-Release Study

To test the controlled release behavior, KGN was dissolved in CMC solution at a concentration of 200.0 μg/mL and mixed with DACNC to form a hydrogel. The high-stiffness hydrogel group was selected as an example. The KGN-containing hydrogel was placed on a rocker at a constant speed (60 rpm) and temperature (40 °C) and immersed in an equal volume of PBS. At predetermined time points, 1 mL of the released solution was collected and replaced with 1 mL of PBS. The concentration of KGN in the released solution was determined using an HPLC system (Agilent HPLC-1260, Infinity, Waldbronn, Germany) with a WondaSil C18-WR column (250 × 4.6 mm, pore size 5 μm, Shimadzu, Kyoto, Japan). A 20 μL sample was injected, and the mobile phase was delivered at a flow rate of 1 mL/min. The mobile phase, consisting of an acetonitrile/water mixture (*v*/*v* = 60/40), was used to extract KGN. After detection with a UV detector at 270 nm, the release curves and release efficiency were determined using the standard curve method.

#### 2.6.2. Histologic Section and Safranin-O Staining

The pellet culture method was adopted to induce the chondrogenesis of RBMSCs. Briefly, 5 × 10^5^ RBMSCs were harvested in 15 mL tubes and centrifuged at 300× *g* for 5 min. The cells were then resuspended in 5 mL of chondrogenic induction medium (Cyagen Biosciences, Guangzhou, China) without TGF-β, containing 10% (*v*/*v*) FBS, 100.0 nM dexamethasone, 50.0 μg/mL ascorbic acid, 6.25 μg/mL insulin, and antibiotic/antimycotic solution and cultured for 21 days to form pellets. KGN at concentrations of 10–150 nM was mixed into the chondrogenic medium. The medium was refreshed every two days. After induction, the pellets were fixed with 4% paraformaldehyde, embedded in paraffin, and sectioned. Safranin-O staining was performed to observe the secreted cartilage matrix. Additionally, the weight of the pellets was recorded.

#### 2.6.3. Sulfated Glycosaminoglycan (sGAG) Content

The content of sGAG in each pellet was detected using the 1,9-dimethyl-methylene blue (DMMB, Sigma, St. Louis, MO, USA) staining assay. First, a standard curve was established using chondroitin-4-sulfate (Sigma, St. Louis, MO, USA) of known concentrations. The cell pellets were lysed to extract sGAG. The extracts were then dyed with DMMB, and the absorbance was measured at 525 nm using a microplate reader (Thermo, Multiskan Go, Waltham, MA, USA). The total sGAG content was determined from the OD values, which were correlated to the standard curve. To eliminate the influence of cell number, the total DNA content was also extracted using the Trizol (Sigma, St. Louis, MO, USA) method and measured with a NanoDrop™ 2000 spectrophotometer (Thermo Fisher Scientific, Waltham, MA, USA). The sGAG content was normalized to the DNA content (μg GAG/μg DNA).

### 2.7. In Vitro Differentiation of RBMSCs Encapsulated in the Hydrogels

#### 2.7.1. Quantitative Real-Time Polymerase Chain Reaction (qRT-PCR) Assay

The hydrogel was incubated in glycine aqueous solution (100 mg/mL) at 37 °C for 1 h to release entrapped cells. The expression of cartilage-related genes, namely aggrecan (ACAN), SOX9, collagen type II (COL2), and collagen type X (COL10), was investigated through qRT-PCR to evaluate the chondrogenic differentiation capacity of RBMSCs in hydrogels with different mechanical properties. The total RNA was extracted using an RNA extraction reagent (OMEGA, Norcross, Georgia, GA, USA) on days 7 and 14 according to the manufacturer’s instructions. The concentration and purity of the extracted RNA were determined spectrophotometrically using a NanoDrop 2000 (Thermo Fisher, USA). Then, 1 μg of total RNA was used for reverse transcription using the 1st Strand cDNA Synthesis SuperMix Kit (Yeasen, Shanghai, China). The synthesized cDNA was then mixed with SYBR Green Master Mix and primers to quantify the gene expression of the chondrogenic-related genes. Gene expression was calculated using the 2^−ΔΔCt^ method. Primer information is provided in Table 1. All experiments were performed in triplicate.

#### 2.7.2. Enzyme-Linked Immunosorbent Assay (ELISA)

The RBMSCs were encapsulated in hydrogels at a density of 2 × 10^5^ cells and incubated in a complete medium at 37 °C and 5% CO_2_. On days 14 and 21, the supernatant was collected from the medium. Then, the levels of COL2 and ACAN secreted into the supernatant were determined using the Enzyme-Linked Immunosorbent Assay (ELISA) Kit (Abcam, Cambridge, MA, USA) according to the manufacturer’s instructions.

#### 2.7.3. RNA Sequence Analysis of RBMSCs

The total RNA was isolated using Trizol reagent (Invitrogen, Carlsbad, CA, USA) and identified with a Bioanalyzer 2100 (Agilent, Santa Clara, CA, USA). The RNA was purified, fragmented into small pieces, and then reverse-transcribed into cDNA. The cDNA was subsequently treated with E. coli DNA polymerase I (NEB, cat.m0209, Ipswich, MA, USA), RNase H (NEB, cat.m0297, Ipswich, MA, USA), and dUTP Solution (Thermo Fisher, cat. R0133, Waltham, MA, USA) to generate U-labeled second-stranded DNAs. The blunt ends of each strand were modified with an A-base, followed by fragment size selection and purification using magnetic beads. The U-labeled second-stranded DNAs were then treated with the UDG enzyme (NEB, cat.m0280, Ipswich, MA, USA) and amplified by PCR. The qualified samples underwent 2 × 150 bp paired-end sequencing (PE150) on an Illumina Novaseq 6000 (LC-Bio Technology Co., Ltd., Hangzhou, China) according to the manufacturer’s protocol. Differentially expressed mRNAs were identified based on a fold change greater than 2 or less than 0.5, with a *p*-value < 0.05. Bioinformatic analysis was conducted using OmicStudio tools at https://www.omicstudio.cn/tool (accessed on 1 October 2024). RT-PCR was performed to validate core gene expression.

### 2.8. In Vivo Test

#### 2.8.1. In Vivo Degradation and Biocompatibility Test

The in vivo degradation and biocompatibility of the hydrogel were tested in Sprague Dawley (SD) rats. In brief, CMC and DACNC were dissolved in PBS at a concentration of 4 wt.%. The high-stiffness group was selected as an example. A volume of 500 μL of the hydrogel was injected subcutaneously into the dorsal area of the SD rat using a syringe with a 21-gauge needle. At designated time points, the rats were sacrificed, and the local area of the injected hydrogel was photographed. Additionally, to evaluate the inflammatory response to the hydrogel in vivo, the skin containing the hydrogel was collected, fixed in 4% paraformaldehyde, and cut into 5 μm thick histologic sections. Hematoxylin and eosin (H&E) staining was performed to observe the inflammatory response.

#### 2.8.2. The Animal Model for Cartilage Defects and Hydrogel Implantation

Thirty-six male New Zealand rabbits were used to evaluate the in vivo cartilage regeneration capacity of the hydrogel. The rabbits were divided into four groups: control (PBS; *n* = 9), hydrogel alone (*n* = 9), hydrogel + RBMSC group (*n* = 9), and hydrogel + RBMSC + KGN group (*n* = 9).

The rabbits were administered routine anesthesia (pentobarbital sodium salt, 30 mg/kg body weight) via intravenous injection through the ear vein. After shaving the hair on the posterior limb and sterilizing it with iodine tincture, a 3-cm longitudinal skin incision was made on the lateral patella to expose the articular surface. A full-thickness cartilage defect with a diameter of 3 mm and a depth of 2 mm was drilled in the center of the trochlear groove. Subsequently, the defects were injected with 100 μL of hydrogel or hydrogel mixture according to the preset groups. The control groups were injected with PBS. Then, the wound was carefully sutured and covered with surgical dressing to prevent infection. An injection of 80,000 U penicillin was administered to each rabbit for 3 days after surgery. Both knees of each rabbit per group were harvested at weeks 4, 8, and 12 (*n* = 3 each) for gross observation and histological evaluation. The harvested samples were assessed using the International Cartilage Repair Society (ICRS) scoring method [23] to evaluate the grade and stage of the articular specimens.

#### 2.8.3. Histological Assessment

To evaluate the extent of tissue formation and reconstruction visually, histological assessments were taken by HE, Toluidine blue (TB), Alcian blue (AB), and safranin-O staining analyses after gross examination. Samples were fixed in 4% formalin for 3 days, decalcified in 15% EDTA for 60 days, and embedded in paraffin. Then, these specimens were cut into 5 μm sections by a paraffin tissue slicer. Afterward, the sections were placed on glass slides and deparaffinized with xylene and ethanol. Histological staining was performed with the above-mentioned dyes. The stained positive area was used to analyze the cartilaginous matrix distribution.

Immunohistochemistry staining was conducted to further evaluate the reconstruction of cartilage. Briefly, sections were dewaxed, dehydrated, and incubated in a methanol solution with 3% hydrogen peroxide for 30 min. After being blocked with 5% goat serum, samples were incubated with Coll-II (Novus, NB600-844, 1:200) for 2 h at 37 °C. Then, they were incubated with a secondary antibody (goat anti-mouse IgG) at 37 °C for 30 min. The chromogenic agent 3,3′-diaminobenzidine (DAB) was used for the chromogenic reaction, then the specific expression of Coll-II was visualized. In addition, we performed immunofluorescence staining on the aggrecan expression in different groups.

### 2.9. Statistical Analysis

All experiments were repeated at least three times. Data were expressed as the mean ± standard deviation (SD). Statistical analysis was conducted with SPSS software 19.0 (IBM, Armonk, NY, USA) using one-way ANOVA and Tukey’s post hoc test. Group differences were considered statistically significant when *p* < 0.05.

## 3. Results

### 3.1. Synthesis and Characterization of Hydrogels

**Figure 2:**
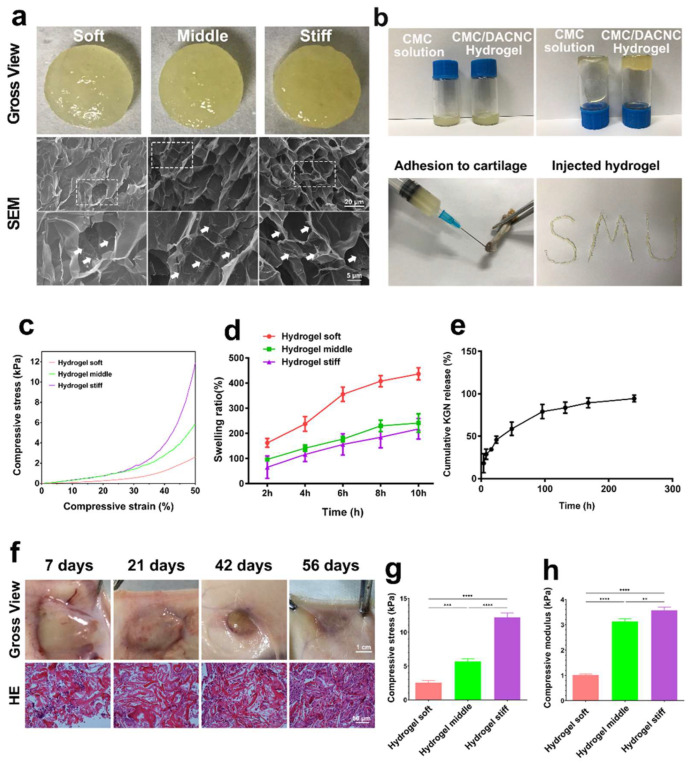
Synthesis and characterization of hydrogels. (**a**) Gross observation and scanning electron microscope of soft, medium, and stiff hydrogels. The dashed box indicates the zoomed area, and the arrows are labeled as “Small fibers”. (**b**) Injectable analysis of the stiff CMC/DACNC-48 (MR = 0.33) hydrogel. (**c**) Representative stress–strain curves of three hydrogel samples. (**d**) Quantification result of swelling ratio. (**e**) KGN-release behavior of the hydrogel in vitro. (**f**) The gross observation of the hydrogel implanted subcutaneously. (**g**,**h**) Quantification result of the compressive Young’s modulus. ** *p* < 0.01, *** *p* < 0.005, **** *p* < 0.001.

The soft, medium, and stiff hydrogels (Figure 2a) all exhibited a translucent appearance and porous structure. Compared with the soft hydrogel, the medium and stiff hydrogels possessed larger quantities of pores due to higher crosslinking density. The stiff hydrogel showed smaller but more homogeneous pores than the medium hydrogel. Small fibers were observed on the surface of the pore walls, which is beneficial for cell adhesion [25]. The CMC/DACNC hydrogels were successfully injected through a narrow 20 G needle, demonstrating shear-thinning behavior (Figure 2b). All three hydrogels exhibited a linear elastic behavior region at low stress and a nonlinear region at high stress (Figure 2c). The swelling ratio of the soft hydrogels was significantly higher than that of the medium and stiff hydrogels, while the difference between the medium and stiff hydrogels was not significant (Figure 2d). As the ratio of CMC/DACNC increased, the compressive strength of the hydrogels increased from 2.53 ± 0.31 kPa to 5.70 ± 0.38 kPa and 12.18 ± 0.65 kPa (Figure 2g). The Young’s modulus was highest for the stiff hydrogel at 3.57 ± 0.13 kPa, significantly higher than the compressive modulus of the medium and soft hydrogels, which were 3.13 ± 0.10 kPa and 1.02 ± 0.04 kPa, respectively (Figure 2h). Gross observation of the hydrogel implanted subcutaneously showed good degradation behavior with minimal inflammatory cell infiltration (Figure 2f).

### 3.2. Sustained KGN-Release Behavior of Hydrogel In Vitro

The drug-release profiles of KGN from the hydrogel were studied in PBS by HPLC (high-performance liquid chromatography) according to a previous study [26]. The results of the in vitro release curves showed that KGN encapsulated in the stiff hydrogel quickly released in 100 h, and after about 180 h, the drug release was up to 90% (Figure 2e).

### 3.3. Cell Viability and Proliferation in Hydrogels

**Figure 3:**
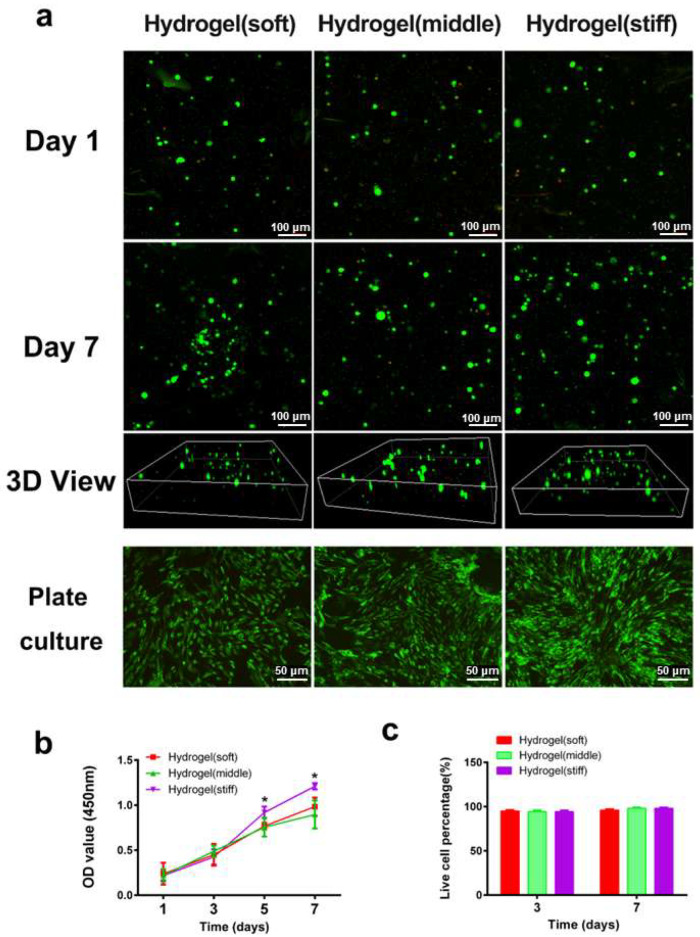
Cell viability and proliferation in hydrogels. (**a**) Representative 3D images of encapsulated cells inside the hydrogel network and plate-cultured cells after 1 day, and 7 days, respectively (green and red dots mean living and dead cells). (**b**) Cell proliferation rate culturing in hydrogels was determined by the CCK-8 assay. (**c**) Representative quantification result of live dead staining. * *p* < 0.05.

The live/dead assay (Figure 3a) and CCK-8 assay (Figure 3b) demonstrated that all three hydrogels exhibited good biocompatibility with minimal cytotoxicity. Fluorescence images showed that the majority of RBMSCs remained viable in all hydrogels on both day 1 and day 7, with few dead cells detected (Figure 3c). Regarding cell proliferation, there were no discernible differences among the three hydrogels on days 1 and 3. Notably, higher cell proliferation rates were observed in the high-stiffness hydrogel compared with the low and medium-stiffness hydrogels on days 5 and 7 (*p* < 0.05), indicating the improved cellular viability of BMSCs in the high-stiffness hydrogel. To further assess cell viability after hydrogel degradation, the hydrogels were dissociated using a glycine aqueous solution to release the encapsulated cells. After culturing for 3 days, the majority of cells remained highly viable with few dead cells observed (Figure 3a), providing additional evidence of the superior cell biocompatibility of the hydrogels.

### 3.4. Cell Differentiation In Vitro

**Figure 4:**
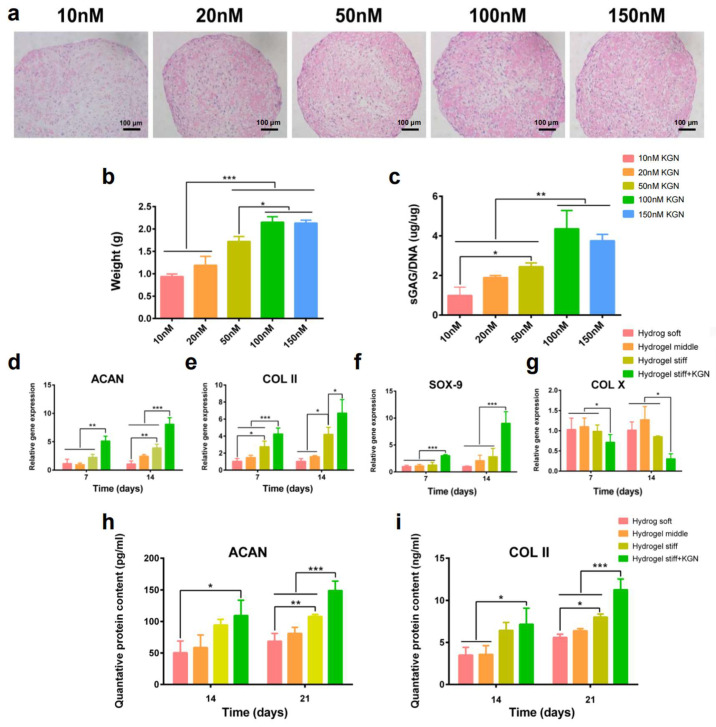
In vitro KGN-induced chondrogenic differentiation. (**a**) The pellet culture method was used to explore the BMSCs’ chondrogenesis abilities with different concentrations of KGN (10–150 nM). Safranin-O staining was performed to observe the secreted cartilage matrix. (**b**,**c**) The weight and sGAG content of the pellets were recorded. (**d**–**g**) RT-PCR results of chondrogenesis related gene expression. (**h**,**i**) Quantified expression of ACAN and collagen-II by the ELISA assay. * *p* < 0.05, ** *p* < 0.01, *** *p* < 0.005.

To investigate the suitable concentration of KGN on the chondrogenic differentiation of RBMSCs, cells were first treated with KGN at different concentrations. After 21 days of induction, the safranin-O staining (Figure 4a) results showed that the secretion of the cartilage matrix increased with the concentration of KGN, which was demonstrated by the gradually deepening dye. Moreover, 100 nM KGN exhibited the most significant amount of cartilage matrix. Furthermore, the size and weight of the formed pellets also demonstrated this tendency (Figure 4b). To further verify this observation, an sGAG detection assay was performed. Results showed that the secretion of sGAG (Figure 4c), which is an important component of the cartilage matrix, was increased with the elevated concentration of KGN while the 100 μL group exhibited the highest amount of sGAG secretion. Thus, these results demonstrate that 100 nM is a suitable concentration of KGN for the chondrogenic differentiation of RBMSCs. The usage of KGN in the following experiments was determined as 100 nM.

RT-PCR and the ELISA assay were used to evaluate the differentiation of encapsulated RBMSCs. Figure 4d–g exhibit the results of the qRT-PCR of four chondrogenesis-related genes, namely aggrecan, SOX-9, collagen-II, and collagen-X. Interestingly, the expression of aggrecan at 14 days and collagen-II at 7 and 14 days was significantly upregulated in the hydrogel stiff group compared with the hydrogel soft and middle groups. Additionally, SOX-9 was also upregulated after 14 days of induction in the hydrogel stiff group. These findings indicate that the hydrogel stiff group with a molar ratio of 0.33 was conducive to cartilage formation. The expression of chondrogenesis-related genes, including aggrecan, SOX-9, and collagen-II, was further significantly upregulated after the addition of KGN, while the expression of the osteogenic marker collagen-X was downregulated. These findings indicate a clear promotion of cartilage differentiation. While gene expression is upregulated, it is ultimately the proteins that exert the functional effects. Given that aggrecan and collagen-II are components of the extracellular matrix primarily found in cartilage, we proceeded to conduct ELISA assays to quantify their expression. As depicted in Figure 4h,i, the levels of ACAN and collagen-II were higher in the hydrogel stiff group, particularly after a 21-day induction period. The most substantial increase in the expression of these two markers was observed in the hydrogel stiff + KGN group, demonstrating a significant upregulation compared with the other groups.

### 3.5. The Mechanism Study of Hydrogel to Enhance Cartilage Regeneration

**Figure 5:**
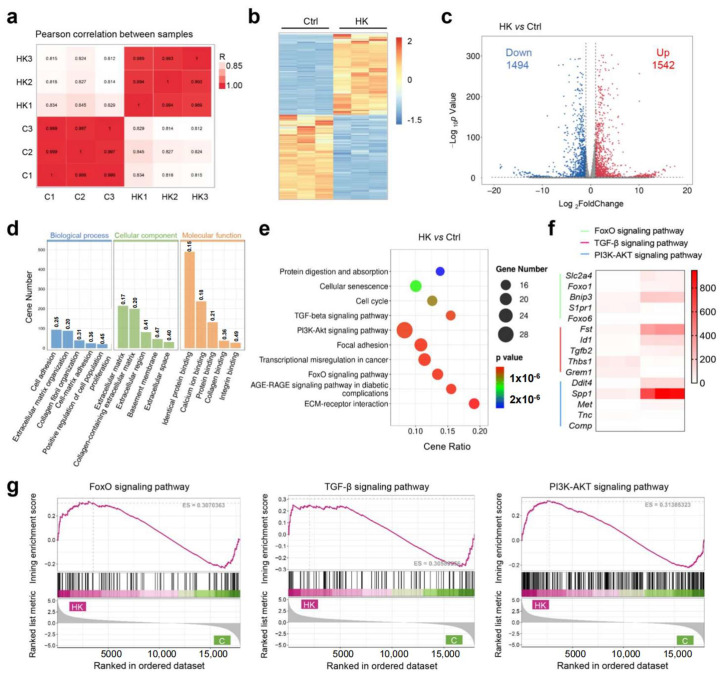
Molecular mechanism study of BMSC differentiation. (**a**) Pearson correlation of the groups. (**b**) Heat map of the total differential expressed genes (DEGs) of the KGN encapsulated hydrogel (HK) vs. the control. (**c**) Volcano plot of DEGs between the HK and control and the corresponding DEG amounts. (**d**) Gene Ontology (GO) analysis of enriched DEGs. (**e**) Kyoto Encyclopedia of Genes and Genomes (KEGG) pathway analysis of DEGs. (**f**) Heat map of the highest differentially expressed genes. (**g**) Gene set enrichment analysis (GSEA) analysis of significantly different pathways.

To investigate the potential molecular mechanisms of hydrogel to enhance cartilage formation, we conducted RNA sequencing (RNA-Seq) to analyze the gene expression profiles of the treated BMSCs between the KGN-loaded hydrogel and control group. The gene expression across all samples was consistent, and met the requirements for subsequent experiments (Figure S1). Pearson correlation analysis demonstrated uniform consistency between the two groups (Figure 5a and Figure S2). The heat map and volcano plot both showed a large number of significantly differential expressed genes (DEGs), among them 1542 were upregulated and 1494 were downregulated in the HK group compared with the control (Figure 5c). For the gene enrich analysis, Gene Ontology (GO) analysis demonstrated that the most significant enriched biological processes were cell adhesion, extracellular matrix organization, collagen fibril organization, proliferation, and cell adhesion. Cellular component enrich analysis showed extracellular matrix was involved during hydrogel regulated cartilage differentiation, and molecular function analysis showed that protein binding, collagen binding, and integrin binding were involved (Figure 5d). As is widely known, the cartilage differentiation process requires the secretion of collagen and polysaccharides, thereby activating extracellular matrix metabolism and protein secretion processes. Our results of the GO analysis proved that a KGN-loaded hydrogel can promote cartilage differentiation via activating extracellular matrix metabolism. The KEGG pathway enrichment results demonstrated cell proliferation, and differentiation-related pathways, such as the TGF-β pathway, FOXO pathway, and PI3K-AKT pathway, were involved (Figure 5e). Gene set enrichment analysis (GSEA) further confirmed that all three pathways were activated in the hydrogel group (Figure 5g). To clarify which genes were significantly regulated, a heat map of the top 5 changed genes of the three pathways was created (Figure 5f). FOXO1 is implicated in cartilage and bone homeostasis, affecting the differentiation and function of chondrocytes and osteoblasts. TGF-β2 is a well-known member of the TGF-β family of cytokines, which have the powerful function of promoting cartilage formation. It plays a role in maintaining bone density and cartilage integrity. The SPP1 gene encodes osteopontin (OPN), which contributes to cartilage homeostasis and repair. The PPI (protein–protein interaction) network was generated using STING analysis, and the core protein was identified. Two gene clusters were identified, which are associated with the extracellular matrix and cell differentiation (Figure S3). Core 20 proteins were identified with Cytoscape (version 3.10.3), which showed cartilage regeneration related proteins such as TGF-β and collagen were included (Figure S4). Furthermore, the enrichment of transcript factors is demonstrated in Figure S5. To confirm the results of gene sequencing, RT-PCR was performed to detect the key gene expression between two groups. The results from Figures S6–S9 demonstrated a strong consistency between the expression of key genes, such as tgfb2, spp1, foxo1, bnip3, and the sequencing data, indicating that the results are reliable. The above results further validate the function of hydrogel to enhance cartilage formation and provide an insight into the molecular mechanisms of its effect in cartilage repair and tissue regeneration.

### 3.6. In Vivo Evaluation of Full-Thickness Cartilage Defect Repair

**Figure 6:**
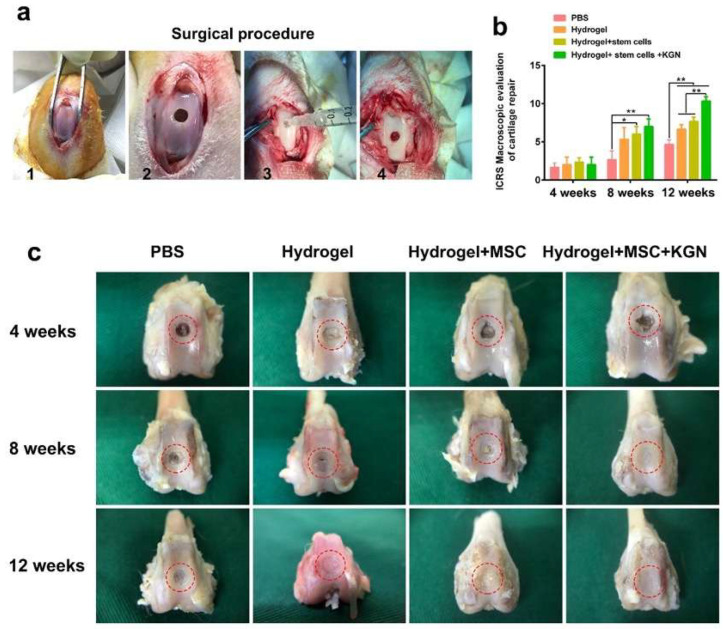
The animal model for cartilage defects and in vivo evaluation of full-thickness cartilage defect repair. (**a**) The surgical procedure of building a full-thickness cartilage defect model. (**b**) The ICRS score of cartilage repair was recorded. (**c**) The representative gross morphology of the cartilage defect site at 4, 8, and 12 weeks. The red circle marks the cartilage defect area. * *p* < 0.05, ** *p* < 0.01.

Macroscopic assessments of cartilage repair were conducted, involving gross observations and evaluations using the ICRS scoring system. Figure 6a outlines the surgical procedure for generating a full-thickness cartilage defect model, which includes exposing the trochlear groove, drilling a uniform hole, injecting materials from each experimental group, and suturing the wound. Due to the notable role of high-stiffness hydrogels in enhancing the chondrogenic differentiation of BMSCs, it was chosen for in vivo experimentation. Four distinct experimental groups were established: hydrogel (H), MSC-encapsulated hydrogel (MH), KGN-MSC-encapsulated hydrogel (KMH), and a control group (PBS). Figure 6c depicts the gross morphology of the cartilage defect site over time. At week 4, there was no significant disparity in neo-cartilage tissue formation among the four groups, with only a slight growth trend observed in the KMH group. By week 8, the KMH group demonstrated the most effective macroscopic regeneration of the cartilage defects, exhibiting complete coverage of the original defect site with neo-cartilage. The H and MH groups also showed significant neo-cartilage growth, although small areas remained uncovered, indicating less efficient regeneration compared with the KMH group. The control group exhibited no significant changes between weeks 4 and 8. Notably, the disparity became more apparent by 12 weeks, with the KMH group showcasing the nearly complete repair of cartilage defects while the H and MH groups still had areas of defects. The control group displayed fibrous tissue filling the defect area instead of mature cartilage at 12 weeks, highlighting the limited self-repair ability of cartilage tissue.

Furthermore, the ICRS score was recorded by three expert orthopedists. As depicted in Figure 6b, there was no discernible difference among the four groups at 4 weeks. However, similar to the gross observations, the ICRS score was significantly higher in the HM and KMH groups at 8 weeks. By 12 weeks, the ICRS scores of the H, HM, and KMH groups were all notably higher than the control group, with the KMH group achieving the highest scores at both 8 and 12 weeks.

**Figure 7:**
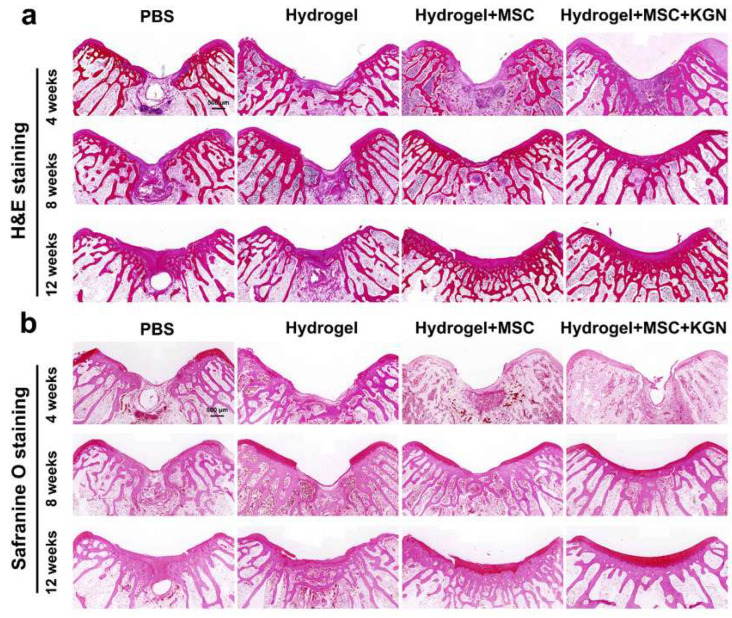
Histological evaluations of the cartilage defect treated with different hydrogels for 4 weeks, 8 weeks, and 12 weeks. (**a**) HE staining for the evaluation of full-thickness cartilage defect repair in the representative image at 4, 8, and 12 weeks; (**b**) safranin-O staining for the evaluation of full-thickness cartilage defect repair in the representative image at 4, 8, and 12 weeks.

HE staining, Toluidine blue (TB) staining, safranin-O (SO) staining, Alician blue (AB) staining, and immunohistochemistry (IHC) for collagen-II were also used to assess the quality of tissue repair. Histological analysis using HE and SO staining revealed substantial differences in cartilage repair among the experimental groups over the 4 to 12-week period (Figure 7a,b). In the control group, persistent subchondral bone defects were observed throughout the study. The cartilage defect areas were predominantly replaced with fibrous tissue, accompanied by inflammatory cell infiltration, and lacked well-organized chondrocytes. Conversely, the H and HM groups exhibited a pronounced trend toward healing over time in both the subchondral bone and cartilage tissue. Despite some new cartilage formation, these groups did not achieve complete structural integration. The KMH group demonstrated the most effective repair outcomes, with bone and cartilage integration evident by 4 weeks, significant new cartilage formation by 8 weeks, and mature cartilage development by 12 weeks.

**Figure 8:**
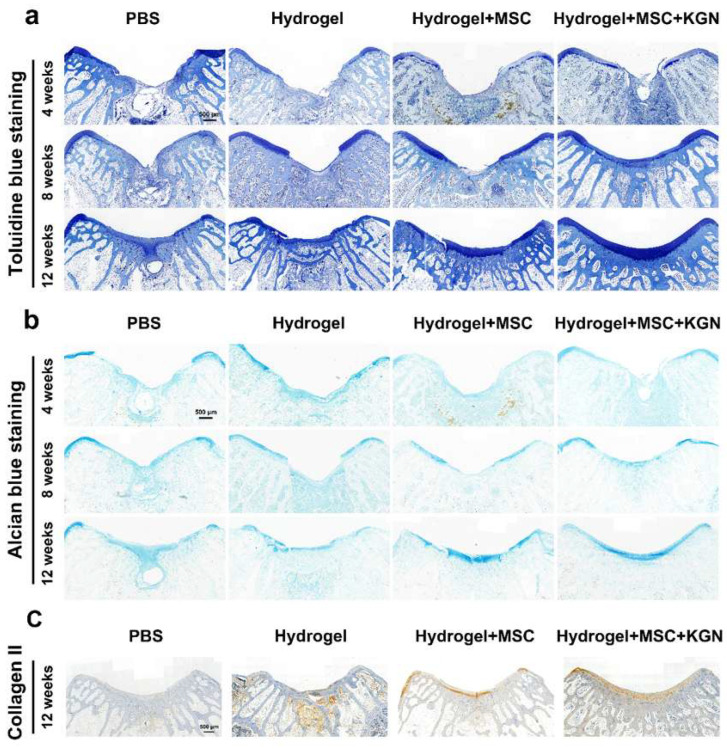
Histological staining for Toluidine blue and Alcian blue of the tissue sections in vivo. (**a**) Toluidine blue staining for evaluation of full-thickness cartilage defect repair in the representative image at 4, 8, and 12 weeks; (**b**) Alcian blue staining for evaluation of full-thickness cartilage defect repair in the representative image at 4, 8, and 12 weeks. (**c**) Immunohistochemistry staining of collagen II at 12 weeks.

Additionally, the presence of a tide mark at 12 weeks indicated a notable enhancement in cartilage tissue repair. TO and AB staining showed a similar tendency, which demonstrated a better extracellular matrix such as glycosaminoglycan or mucopolysaccharide expression in the H, MH, and KMH groups compared with the control group, and most significantly in the KMH group (Figure 8a,b). The quantitative analysis further confirmed this trend (Figure S10). These findings affirm the efficacy of the designed hydrogel in promoting MSC proliferation and differentiation, thus offering a promising approach for facilitating efficient cartilage regeneration. Immunohistochemistry staining of collagen II was additionally performed to assess the collagen fiber content within neo-cartilage tissue. Figure 8c illustrates the superior collagen content observed in the KMH group. To further assess the cartilage matrix content in vivo, ACAN immunofluorescence staining was performed. ACAN immunofluorescence staining showed that the content of ACAN in the newly formed cartilage tissue in the experimental group was significantly higher than that in the control group, indicating the promotion of cartilage regeneration (Figure S11). These findings affirm the efficacy of the designed hydrogel in promoting MSC proliferation and differentiation, thus offering a promising approach for facilitating efficient cartilage regeneration.

## 4. Discussion

Tissue engineering hinges on three fundamental components: stem cells, growth factors, and scaffolds. These components must work synergistically to replicate the physiological environment of native tissues and promote functional regeneration [27]. Injectable cell-loaded hydrogels have garnered significant attention due to their adaptability to irregularly shaped sites and their potential for minimally invasive surgery, consequently, they represent a burgeoning area in cartilage regeneration [28,29,30]. This study developed and characterized a novel injectable CMC/DACNC hydrogel loaded with MSCs and KGN for cartilage repair. The advantages of the constructed hydrogel are as follows: (1) easy formation of an injectable hydrogel through simple mixing without the need for cross-linking agents or irradiation, thus simplifying the preparation process; (2) good biocompatibility and degradability, making it an ideal carrier for stem cells that is capable of maintaining their viability and differentiation potential; (3) superior drug release properties and drug-loading capacity enable it to carry various small molecule or protein drugs; (4) adjustable mechanical properties of the hydrogel by altering the molar ratio of CMC to DACNC, thereby optimizing the mechanical characteristics of the scaffold, supporting the differentiation and secretory activity of mesenchymal stem cells (MSCs), and promoting cartilage repair [31]. These advantages of the hydrogel endow it with significant potential for clinical applications.

The capacity of MSCs to differentiate into a wide range of specialized cell types, coupled with their widespread presence in numerous tissues, renders them a highly advantageous alternative therapeutic for tissue engineering applications [32,33,34]. For MSCs to function effectively, the foremost requirement is the biocompatibility of the carrier, which must also support their long-term self-renewal and differentiation capabilities in vivo. Many materials fall short in biocompatibility due to the necessity for chemically toxic crosslinking agents, photopolymerization, or the inherent cytotoxicity of the base components. In this experiment, we utilized carboxymethyl chitosan and aldehyde-modified cellulose nanocrystals, both natural products exhibiting superior biocompatibility. Their biocompatibility was further evidenced by the minimal inflammatory cell response observed following subcutaneous implantation. Their ability to form a hydrogel through simple mixing and long-term degradation presents significant advantages as MSC carriers. We utilized the hydrogel for the in vitro 3D culture of MSCs, and the results indicated almost no cell death during the culture period. Moreover, the hydrogel with an MR of 0.33 promoted stem cell proliferation, further confirming the hydrogel’s good biocompatibility. MSCs are multipotent cells capable of differentiating into various lineages in response to environmental cues. This necessitates a more precise design of hydrogels that are tailored to the mechanical environment required for the chondrogenic differentiation of MSCs [35,36,37]. To more precisely facilitate the chondrogenic differentiation of MSCs, we adjusted the ratio of CMC to DACNC to obtain hydrogel carriers with varying stiffness. Ultimately, the hydrogel with an MR = 0.33 exhibited the highest mechanical properties. Although its mechanical performance remained significantly lower than that of mature cartilage, which has a reported high modulus of up to 100 MPa [38], it demonstrated the most pronounced differentiation-promoting effects compared with other MR ratios. Some of the literature indicates that factors such as matrix stiffness and viscoelasticity can play a crucial regulatory role in the biological function direction of MSCs [39]. In 2006, Engler et al. reported the dominant role of different extracellular matrix hardness in the differentiation direction of MSCs. A harder matrix plane is conducive to cell adhesion, forming more effective cell-extracellular matrix anchoring, known as adhesion spots [40]. Elosegui Artola and colleagues also found that a harder matrix could increase the cytoskeletal tension by promoting cell extension, resulting in an increase in nuclear pore diameter, which is more conducive to the nuclear-cytoplasmic shuttle of YAP and ultimately affects the direction of cell differentiation [41]. Meanwhile, matrix degradability is also an important factor for MSC migration [42,43]. Mooney’s team first showed that the hydrogels’ viscoelasticity plays an important role in regulating the direction of the MSCs’ extension and differentiation. Their research proposed that viscoelasticity is an inherent attribute of natural tissue and the extracellular matrix, and hydrogels with this attribute can significantly change the response mode of cells and make MSCs show a dose-related response similar to the action of chemical factors [44]. It is worth noting that although the mechanical properties of ideal hydrogels are intended to closely approximate those of cartilage, enhancements in mechanical performance often compromise other characteristics such as injectability and biocompatibility. This trade-off arises from the potential increase in toxicity associated with higher degrees of cross-linking in the hydrogels. Hence, our approach involves designing a hydrogel that not only creates a conducive microenvironment for stem cell differentiation and proliferation, but also facilitates drug loading to induce cartilage regeneration, rather than directly replacing damaged cartilage.

KGN, a small molecule substance identified in 2012, plays a pivotal role in promoting the proliferation and chondrogenic differentiation of MSCs by modulating signaling pathways like core-binding factor β subunit (CBFβ)—runt-related transcription factor 1 (RUNX1) [21]. To improve the solubility, stability, retention time, and controlled release of KGN in the joint, extensive studies have chosen hydrogels as drug delivery vehicles for KGN [45,46,47,48]. Li et al. described the utilization of a temperature-sensitive hydrogel for co-delivering KGN and BMSCs to repair cartilage defects. KGN was physically incorporated into the hydrogel, leading to the release of approximately 42.2% of KGN over 196 h [46]. Xu et al. employed host–guest interactions in the preparation of a photo-crosslinked hydrogel consisting of acrylated β-CD and methacrylated gelatin. KGN was loaded into the hydrophobic cavity of β-CD, enabling stable and sustained release over 28 days [45]. Our study demonstrated that the designed CMC/DACNC hydrogel possessed excellent KGN controlled release character, which could ensure long-term stimulation during the cartilage regeneration process. In vitro experiments demonstrated that KGN-loaded hydrogels effectively enhance the differentiation of BMSCs into cartilage. To further investigate the underlying mechanism, we performed total RNA transcriptome sequencing to examine the impact of the hydrogel on the BMSC differentiation process. Notably, key cartilage differentiation pathways, including TGF-β, FOXO, and PI3K-AKT, were significantly upregulated in the hydrogel group. These findings provide additional confirmation of the hydrogel’s role in promoting cartilage regeneration. The in vivo results demonstrated that the KGN-loaded hydrogel group exhibited a glossy and smooth membrane, closely resembling native cartilage. In contrast, the control group displayed irregular and depressed regenerated tissues after 12 weeks, which were distinguishable from the surrounding cartilage. Thus, it is believed that the constructed CMC/DACNC hydrogel could extend the half-lives of KGN and alleviate their negative effects by confining them within the matrix, thus decreasing rapid proteolysis, burst release, and undesired diffusion.

For this study, we utilized 46 rabbits, including 36 eight-week-old rabbits for the animal model and 10 neonatal rabbits, for the isolation of bone marrow-derived mesenchymal stem cells (BMSCs). This ensured that all MSCs were sourced from bone marrow to minimize individual variability. Although MSCs often display significant growth variability, standardized protocols and stringent quality control measures in this study resulted in minimal standard deviations, ensuring reliable experimental outcomes. Key measures included standardized cell sourcing and processing, uniform preparation of CMC/DACNC hydrogels with a precise molar ratio (MR = 0.33) to guarantee consistent mechanical and chemical properties, and strict control over the environmental conditions (e.g., temperature, humidity, CO_2_ levels) and reagents during cell culture and experiments.

We selected rabbit bone marrow-derived mesenchymal stem cells (RBMSCs) from neonatal rabbits as the primary cell source for our in vitro experiments, without involving other cell lines. This choice was based on several considerations: neonatal animal-derived MSCs typically exhibit higher proliferation and differentiation potential, particularly in cartilage regeneration, providing a robust cellular foundation for the study. The rabbit model is widely used in cartilage defect research due to its high experimental reproducibility and clinical relevance, effectively simulating human cartilage repair processes. Additionally, using animal-derived MSCs avoids ethical concerns associated with human stem cells while facilitating standardized laboratory procedures.

In this study, we selected 4-week-old neonatal New Zealand rabbits as the source of mesenchymal stem cells (MSCs) because neonatal animal-derived MSCs typically exhibit higher activity, with enhanced proliferation and differentiation potential, making them particularly suitable for tissue engineering applications such as cartilage regeneration [49]. When neonatal rabbit MSCs were cultured in KGN-loaded hydrogels, we observed significant chondrogenic differentiation capacity. RNA-sequencing analysis further revealed the upregulation of cartilage-related signaling pathways (e.g., TGF-β, FOXO, and PI3K-AKT), confirming their high regenerative responsiveness. Thus, the choice of neonatal rabbit MSCs was justified not only by their superior proliferation and differentiation potential, but also by their remarkable regenerative efficacy, providing an optimal cellular foundation for the experiments.

Although this study did not directly investigate MSCs from alternative sources (e.g., adipose tissue), the existing literature suggests that MSCs from different tissues vary in differentiation potential and microenvironmental responsiveness [50]. For instance, BM-MSCs exhibit strong chondrogenic differentiation capacity, whereas AT-MSCs and DT-MSCs show comparatively lower potential. Therefore, using MSCs from other sources may require the re-optimization of hydrogel formulations and experimental conditions to ensure comparable cartilage regeneration outcomes. Future studies could explore the effects of different MSC sources to further validate their applicability.

While this study demonstrates the hydrogel’s efficacy in cartilage repair, future work should evaluate immune cell infiltration dynamics (e.g., macrophages, mast cells) in the peri-cartilage region, as emerging evidence suggests their critical role in modulating inflammation-mediated tissue remodeling [51,52].

## 5. Conclusions

In summary, we successfully developed an injectable CMC/DACNC hydrogel by simply mixing CMC with DACNC and investigated its potential for cartilage repair. This hydrogel exhibited good biocompatibility, maintaining the activity and self-repair ability of MSCs over an extended period. Additionally, it effectively loaded the bioactive drug KGN, achieving controlled release. By adjusting the MR between CMC and DACNC, we determined that a high-stiffness hydrogel with an MR of 0.33 was the most suitable for cartilage induction. The in vitro study confirmed that the KGN-loaded hydrogel promotes cartilage differentiation by activating the TGF-β, FOXO, and PI3K-AKT pathways. Furthermore, we utilized the CMC/DACNC hydrogel to co-load MSCs and KGN, observing optimal cartilage regeneration in vivo. In conclusion, this injectable hydrogel presents a novel option with sufficient biosafety and efficacy for cartilage regeneration and holds great potential for clinical minimally invasive treatment.

## Figures and Tables

**Table 1:** Primers used in RT-PCR.

Gene	Forward Primer Sequence (5′-3′)	Reverse Primer Sequence (3′-5′)
ACAN	CTCAGACCTCGACAACGCAT	TAGTTGGGCAGCGAGACCTT
Collagen-II	TGCAGGAGGGGAAGAGGTAT	TCCTTTCTGCCCCTTTGGTC
Sox-9	GGCTCCGACACCGAGAATA	GTCTCCAGAGCTTCCCGAGG
Collagen-X	GGCATAAAAGGCCCACAACC	TTGGTCCTCTCTCCCCTTGT
GAPDH	AGAGCACCAGAGGAGGACG	TGGGATGGAAACTGTGAAGAG

## Data Availability

The original contributions presented in this study are included in the article/Supplementary Materials. Further inquiries can be directed to the corresponding authors.

## Supplementary Materials

The following supporting information can be downloaded at: https://www.mdpi.com/article/10.3390/bioengineering12050434/s1, Figure S1: Gene expression violin; Figure S2: Sample PCA gene; Figure S3: PPI network; Figure S4: PPI core 20 proteins; Figure S5: Transcript Factor family barplot; Figure S6: Foxo1 express validation; Figure S7: Spp1 express validation; Figure S8: Tgfb2 express validation; Figure S9: Bnip3 express validation; Figure S10: Quantification of tissue sections; Figure S11: ACAN immunofluorescence staining; Figure S12: Morphological and functional characterization of isolated RBMSCs.
