# Supplementary figures and images for: An Injectable Kartogenin-Incorporated Hydrogel Supports Mesenchymal Stem Cells for Cartilage Tissue Engineering
Source: Bioengineering (Basel). 2025 Apr 22;12(5):434. doi: 10.3390/bioengineering12050434 (PMC12108833; doi:10.3390/bioengineering12050434)

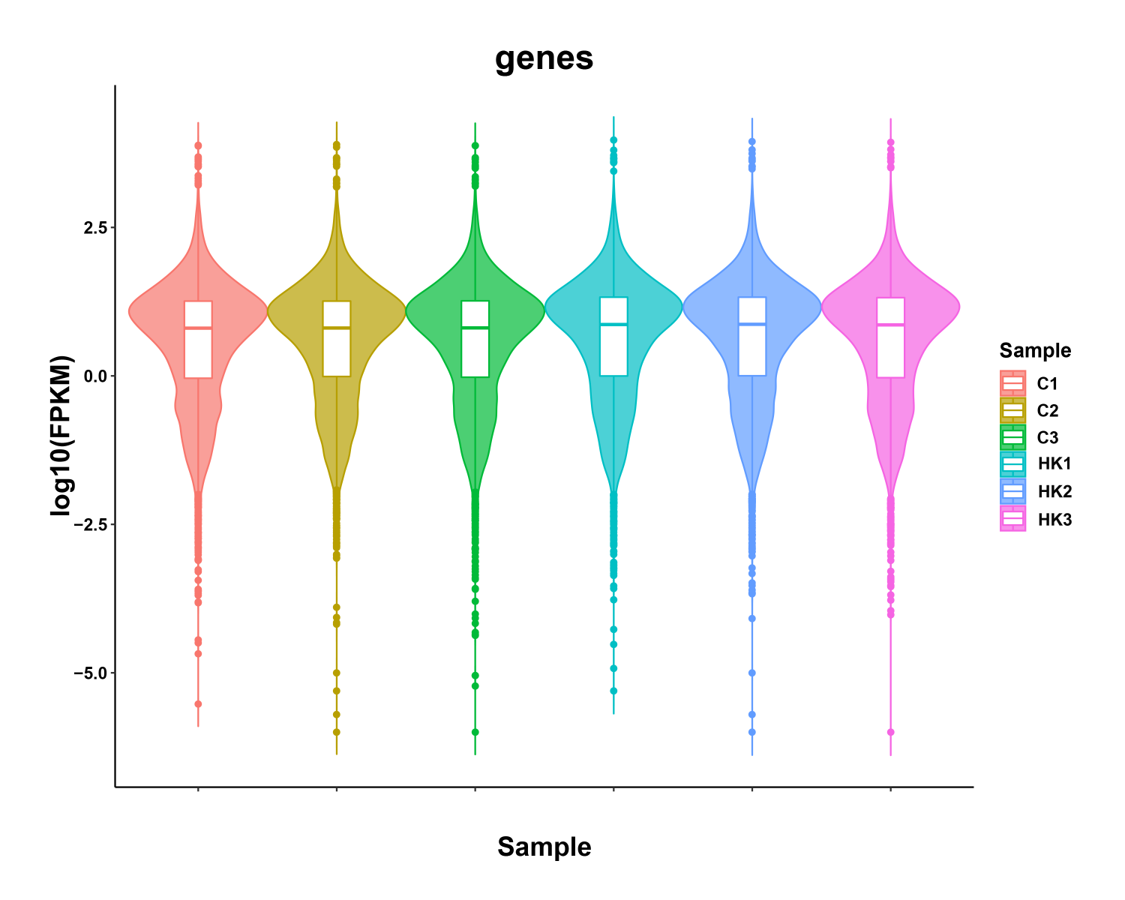

Supplement: Supplementary file 1 [file bioengineering-12-00434-s001.zip › Figure S1 gene_expression_violin.tif]

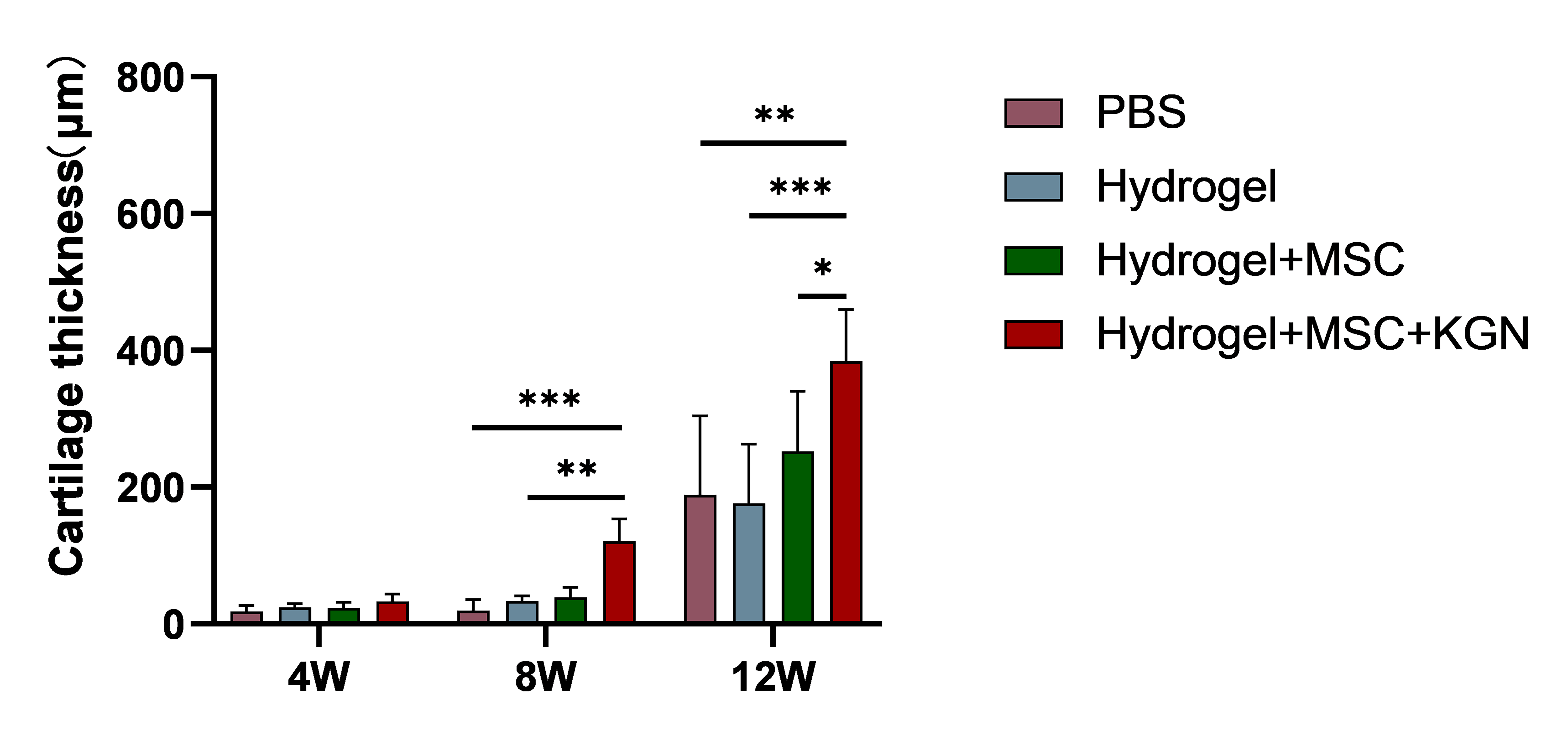

Supplement: Supplementary file 1 [file bioengineering-12-00434-s001.zip › Figure S10 quantification of tissue sections.tif]

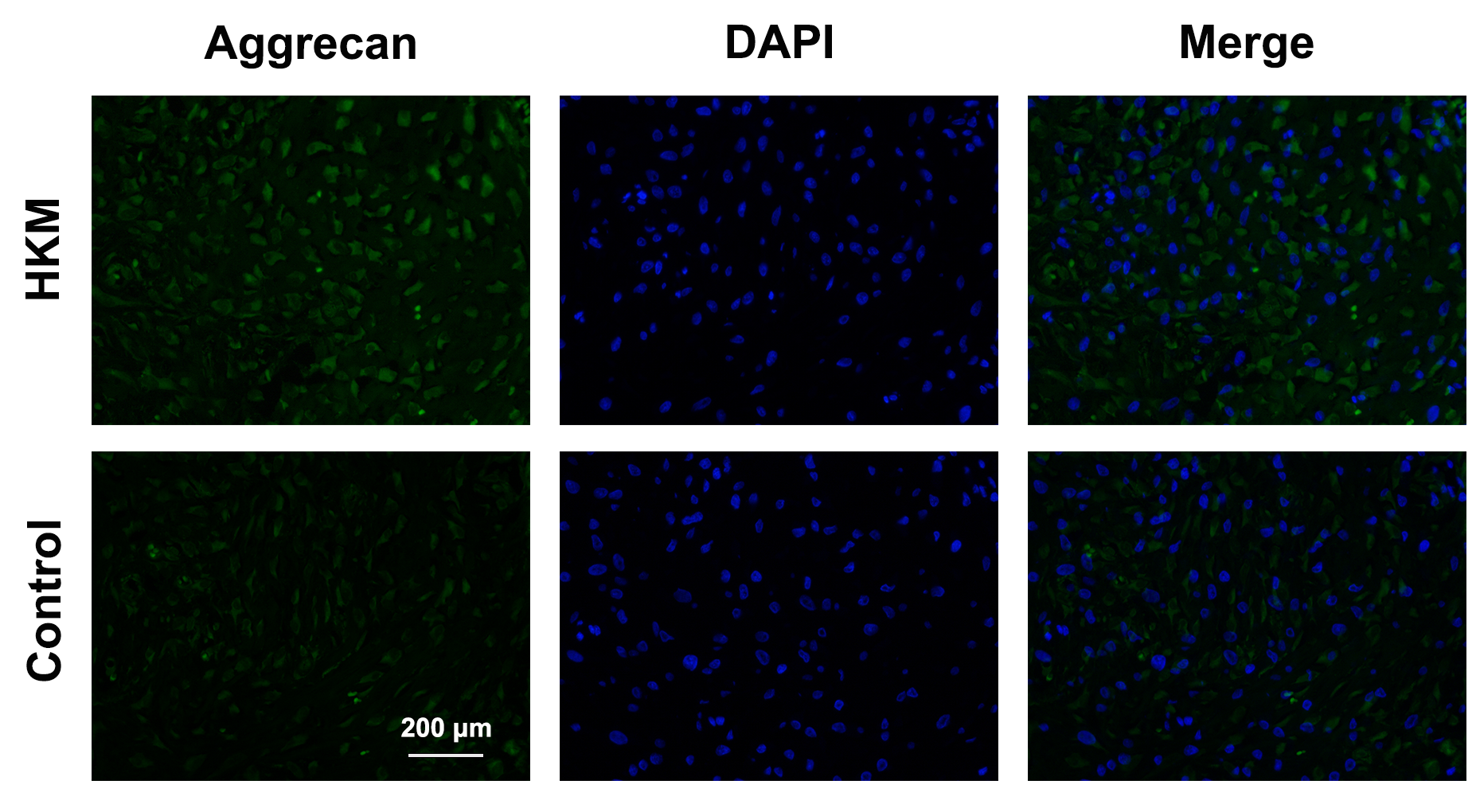

Supplement: Supplementary file 1 [file bioengineering-12-00434-s001.zip › Figure S11 ACAN immunofluorescence staining.tif]

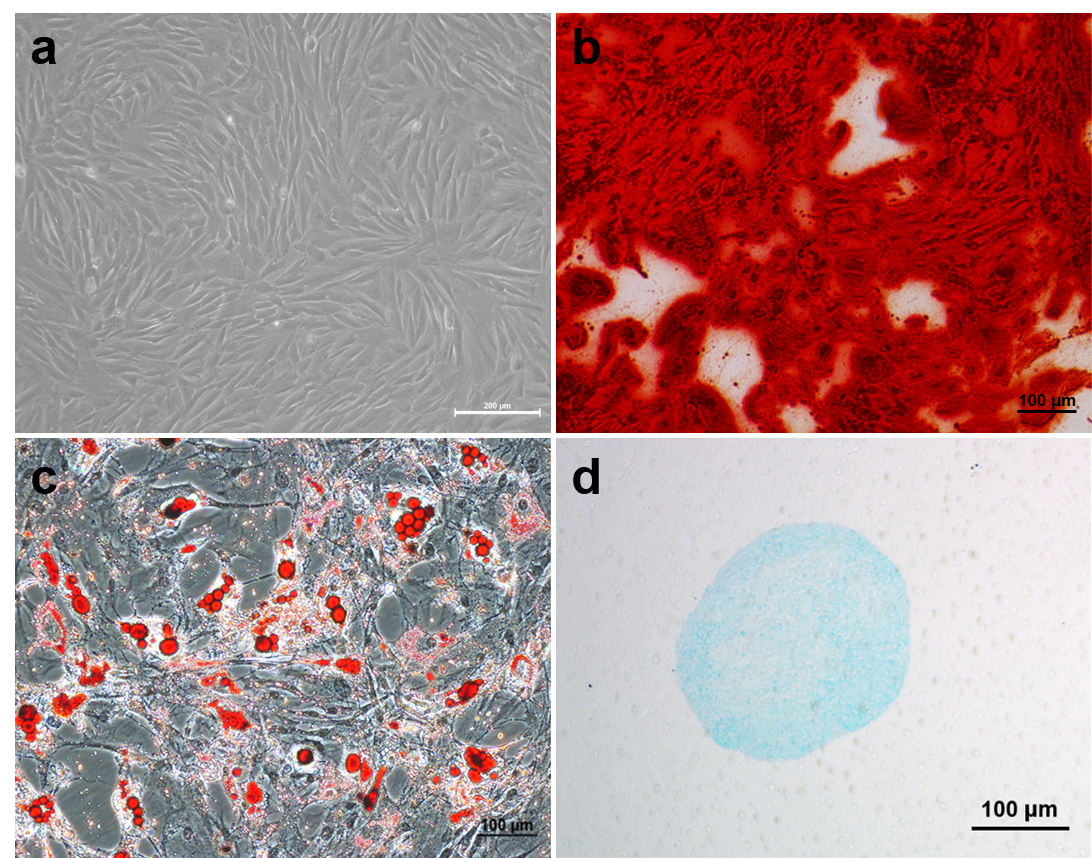

Supplement: Supplementary file 1 [file bioengineering-12-00434-s001.zip › Figure S12 morphological and functional characterization of isolated RBMSCs.tif]

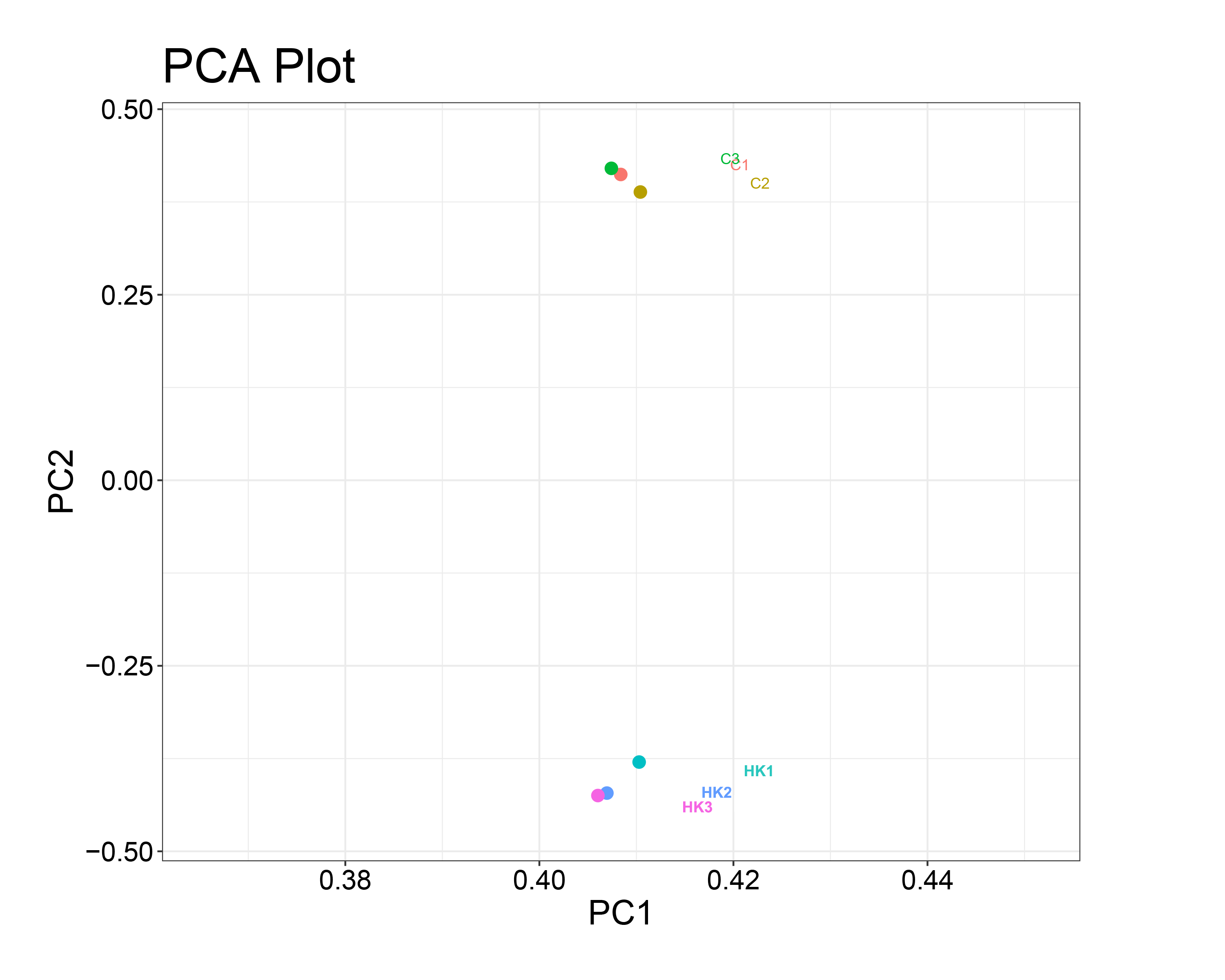

Supplement: Supplementary file 1 [file bioengineering-12-00434-s001.zip › Figure S2 sample_PCA_gene.tif]

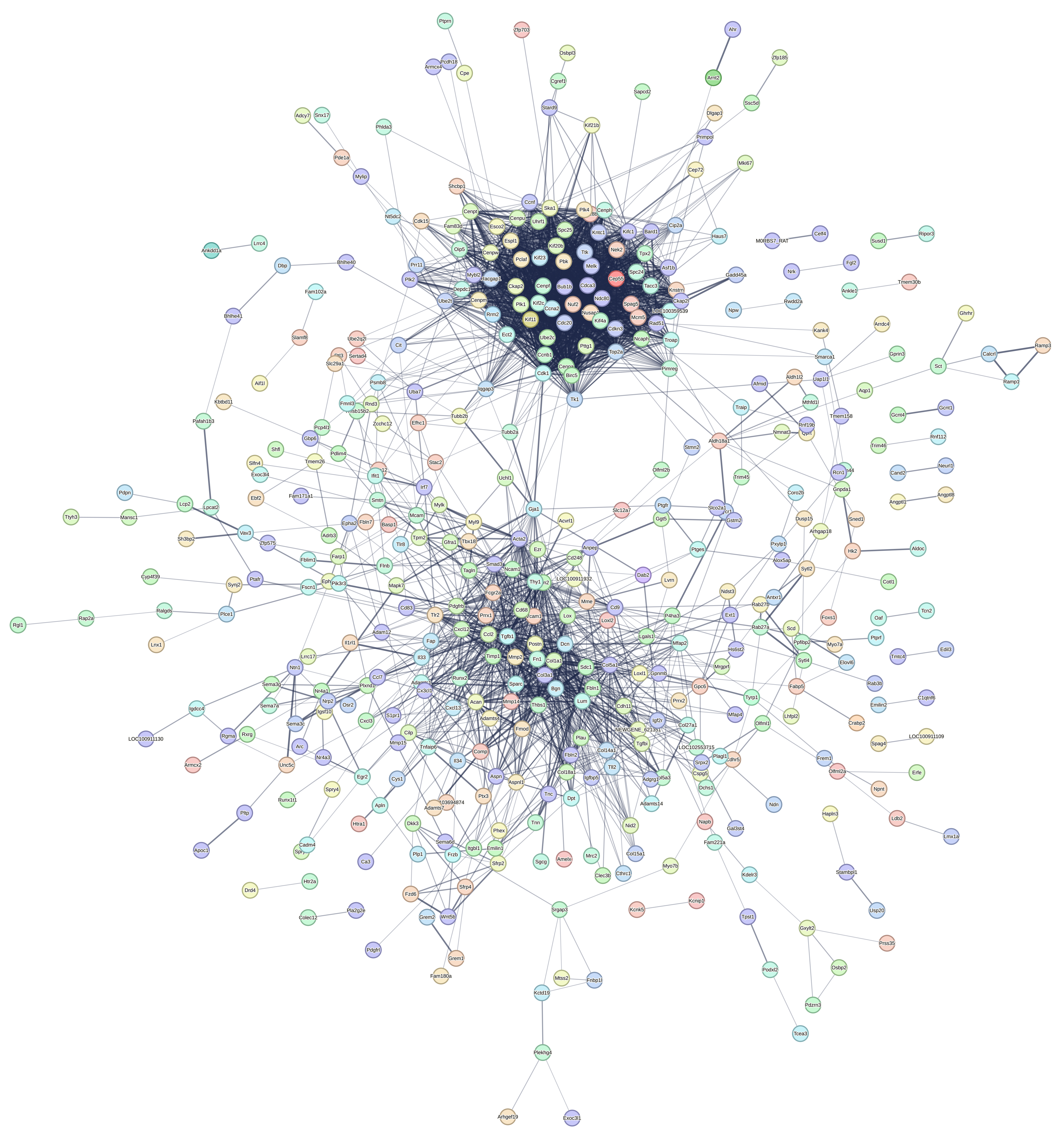

Supplement: Supplementary file 1 [file bioengineering-12-00434-s001.zip › Figure S3 PPI network.tif]

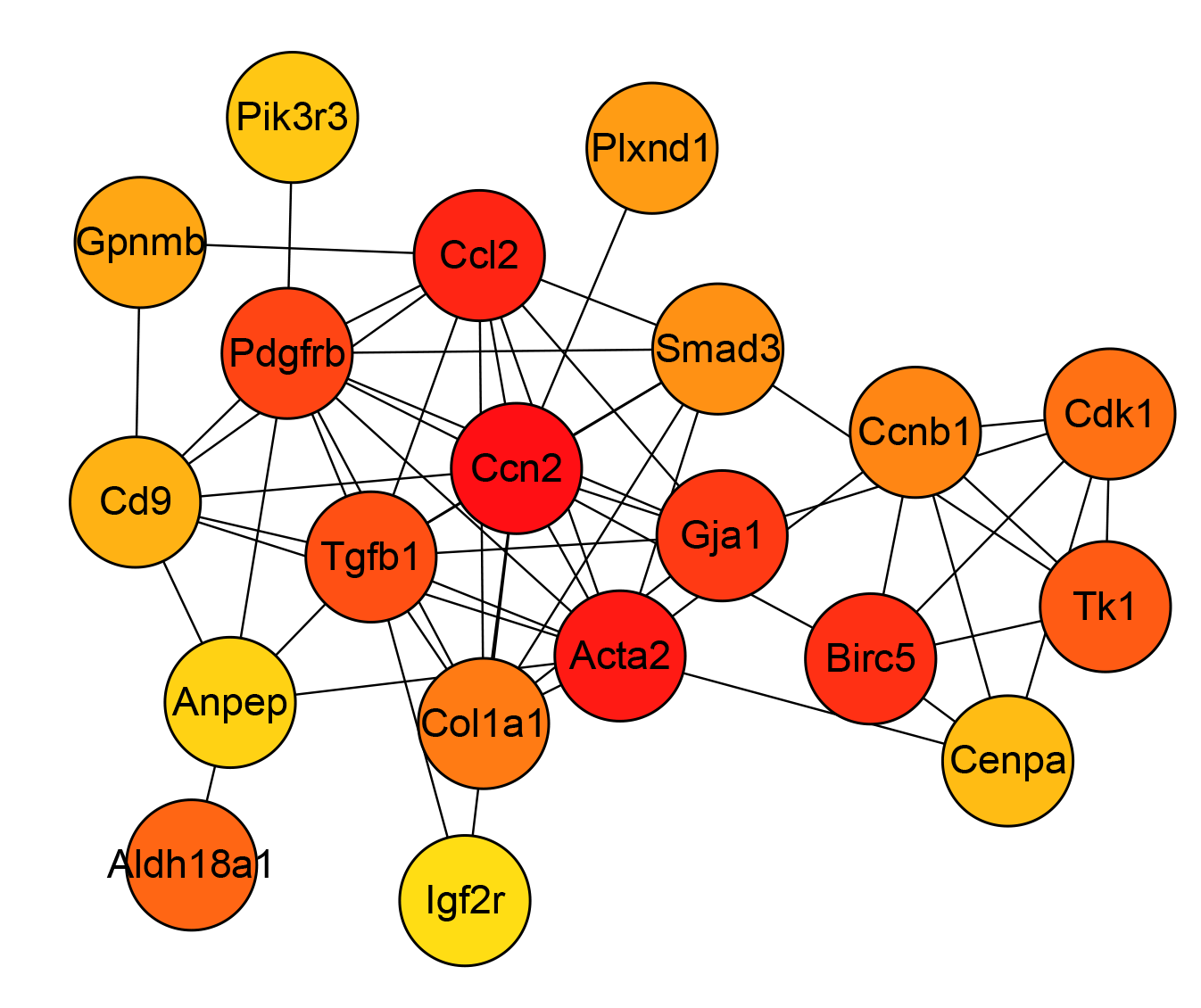

Supplement: Supplementary file 1 [file bioengineering-12-00434-s001.zip › Figure S4 PPI core 20 proteins.tif]

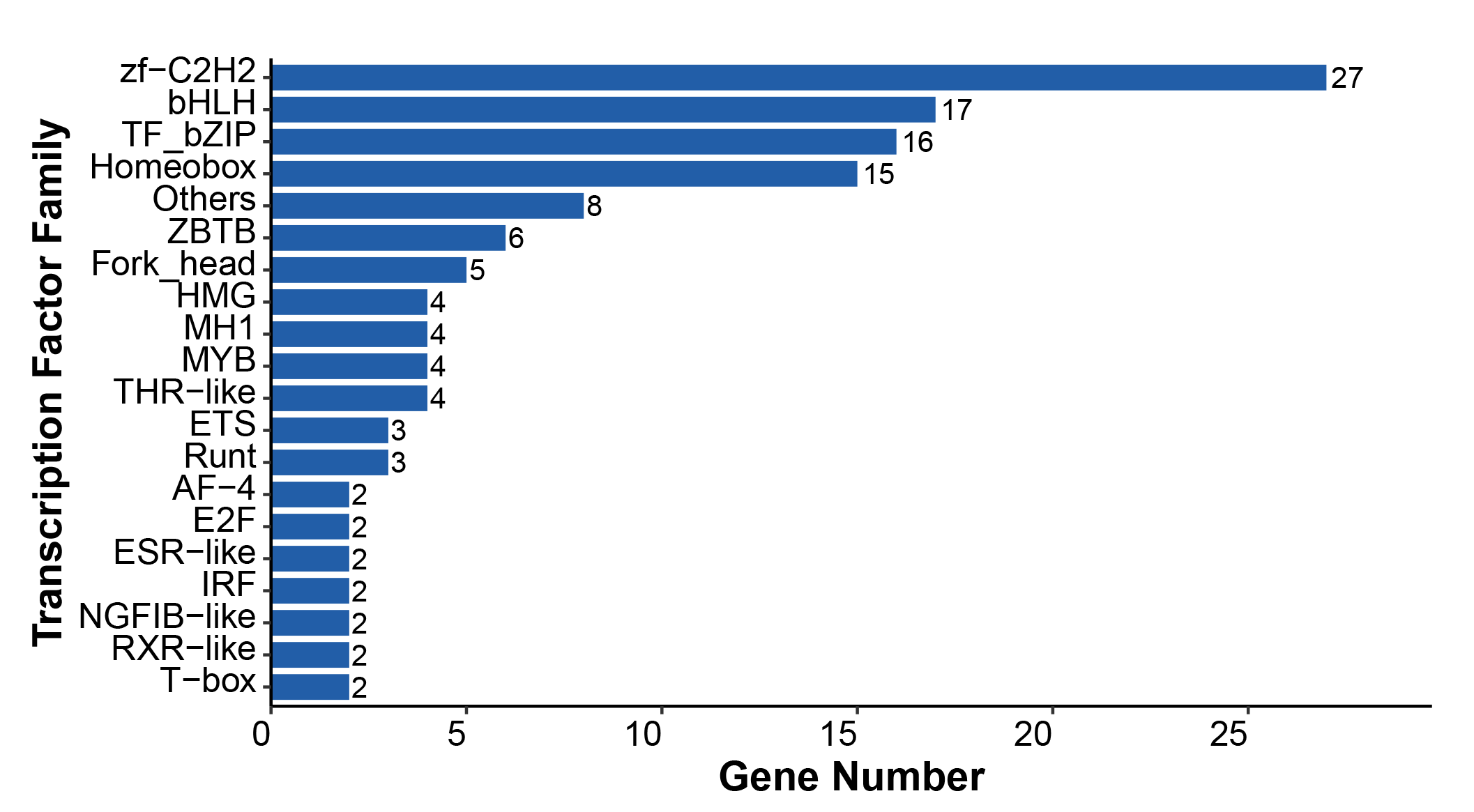

Supplement: Supplementary file 1 [file bioengineering-12-00434-s001.zip › Figure S5 Ttranscript Factor family barplot.tif]

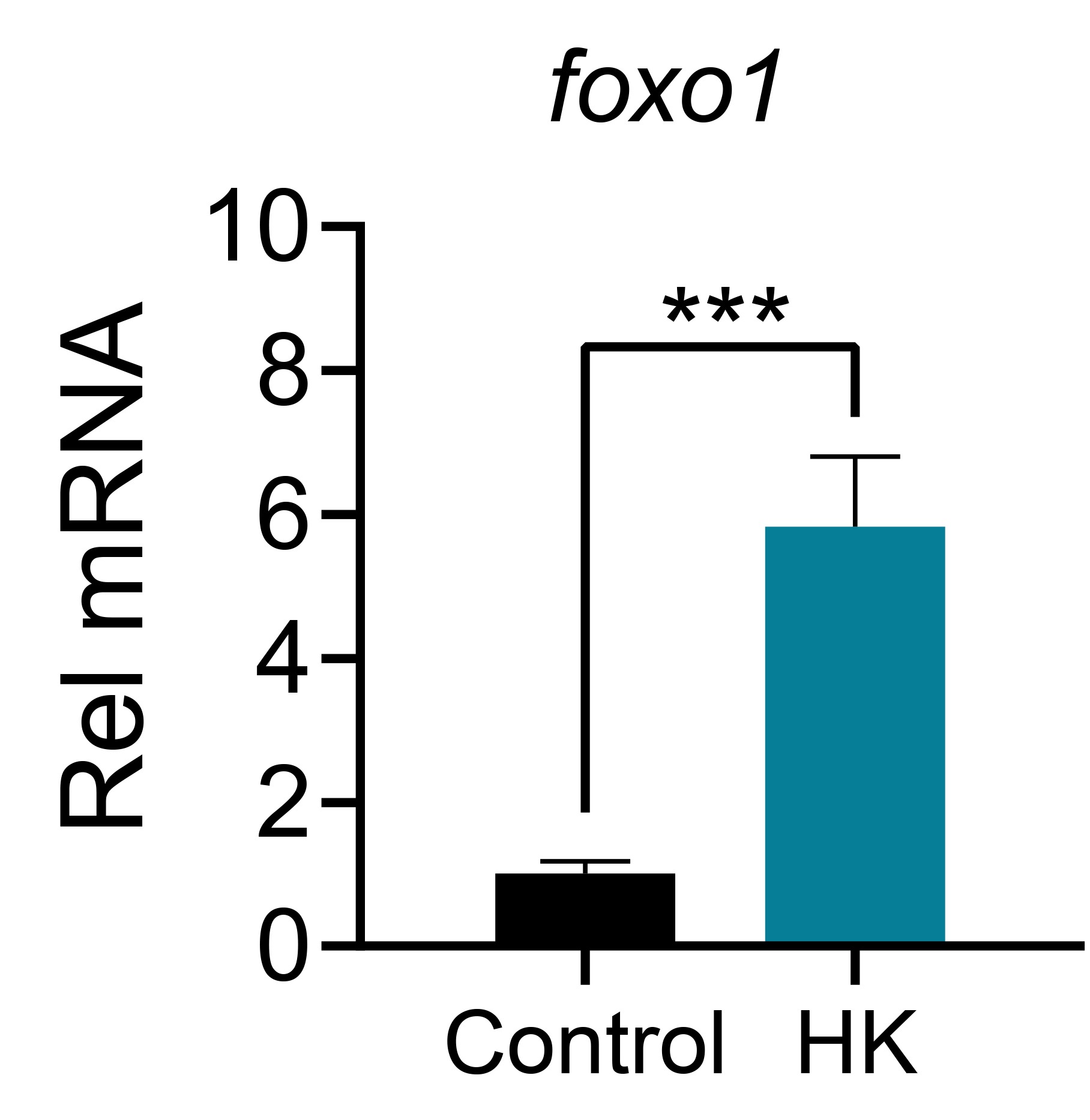

Supplement: Supplementary file 1 [file bioengineering-12-00434-s001.zip › Figure S6 foxo1 express validation.tif]

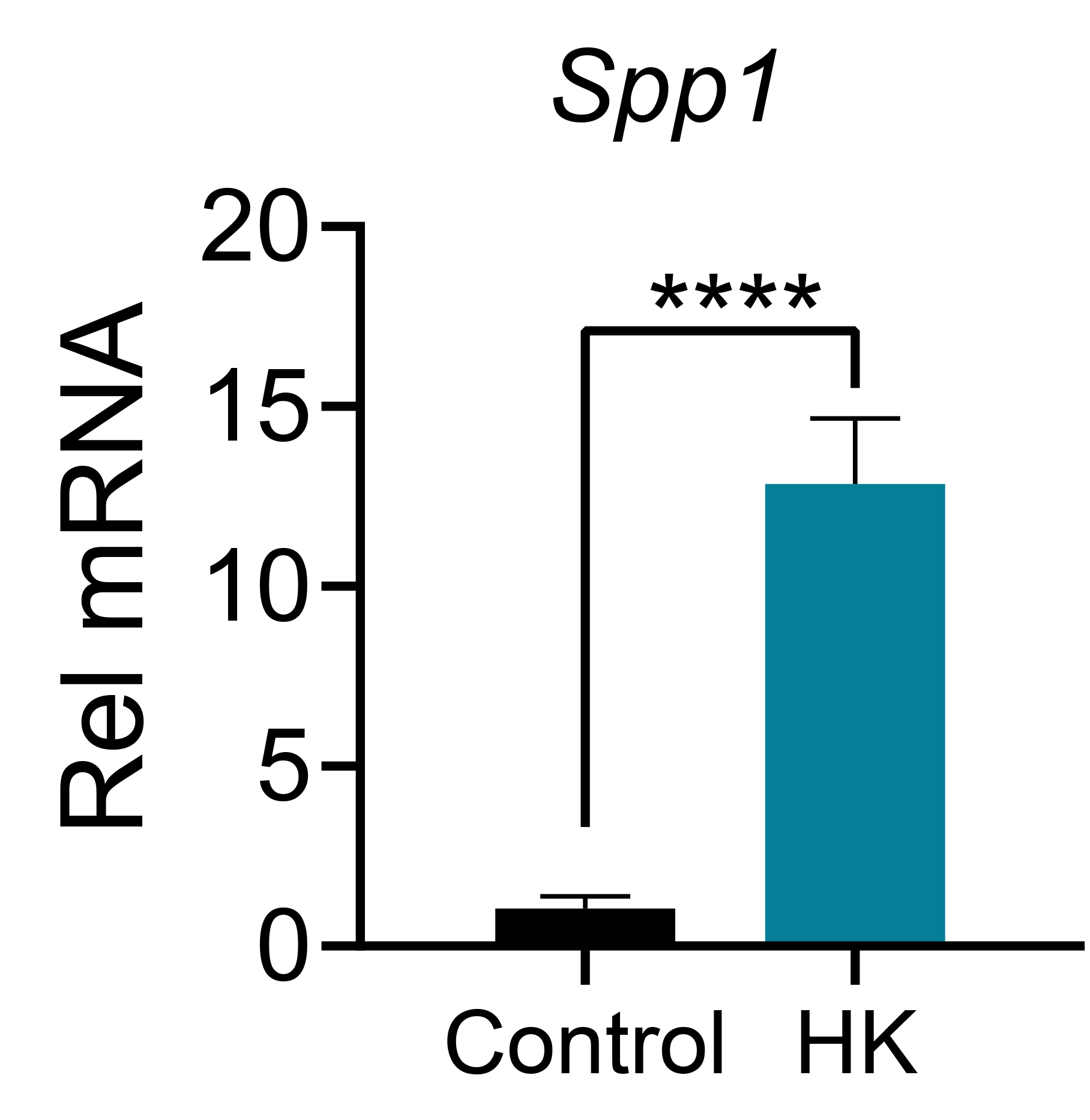

Supplement: Supplementary file 1 [file bioengineering-12-00434-s001.zip › Figure S7 Spp1 express validation.tif]

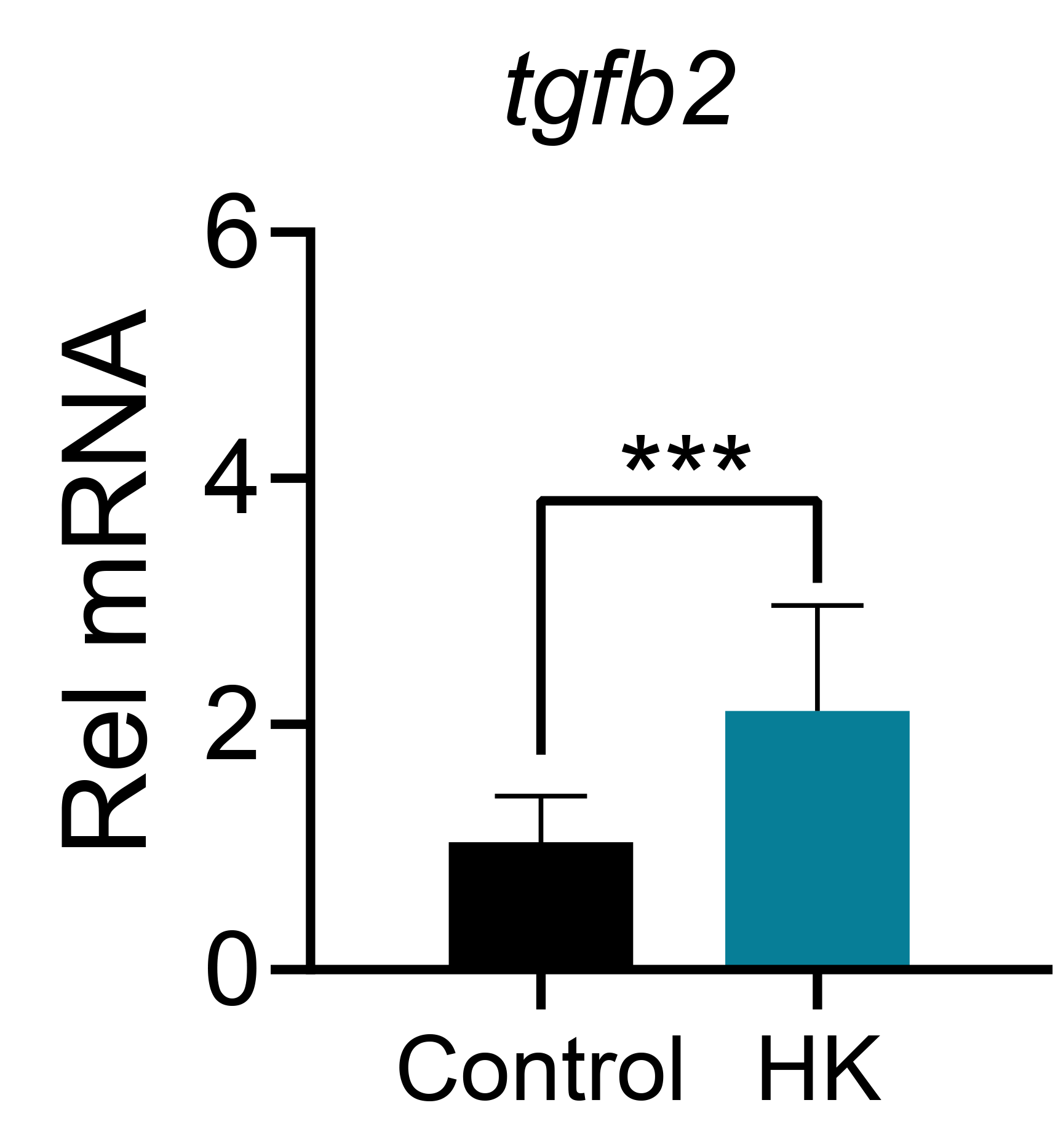

Supplement: Supplementary file 1 [file bioengineering-12-00434-s001.zip › Figure S8 tgfb2 express validation.tif]

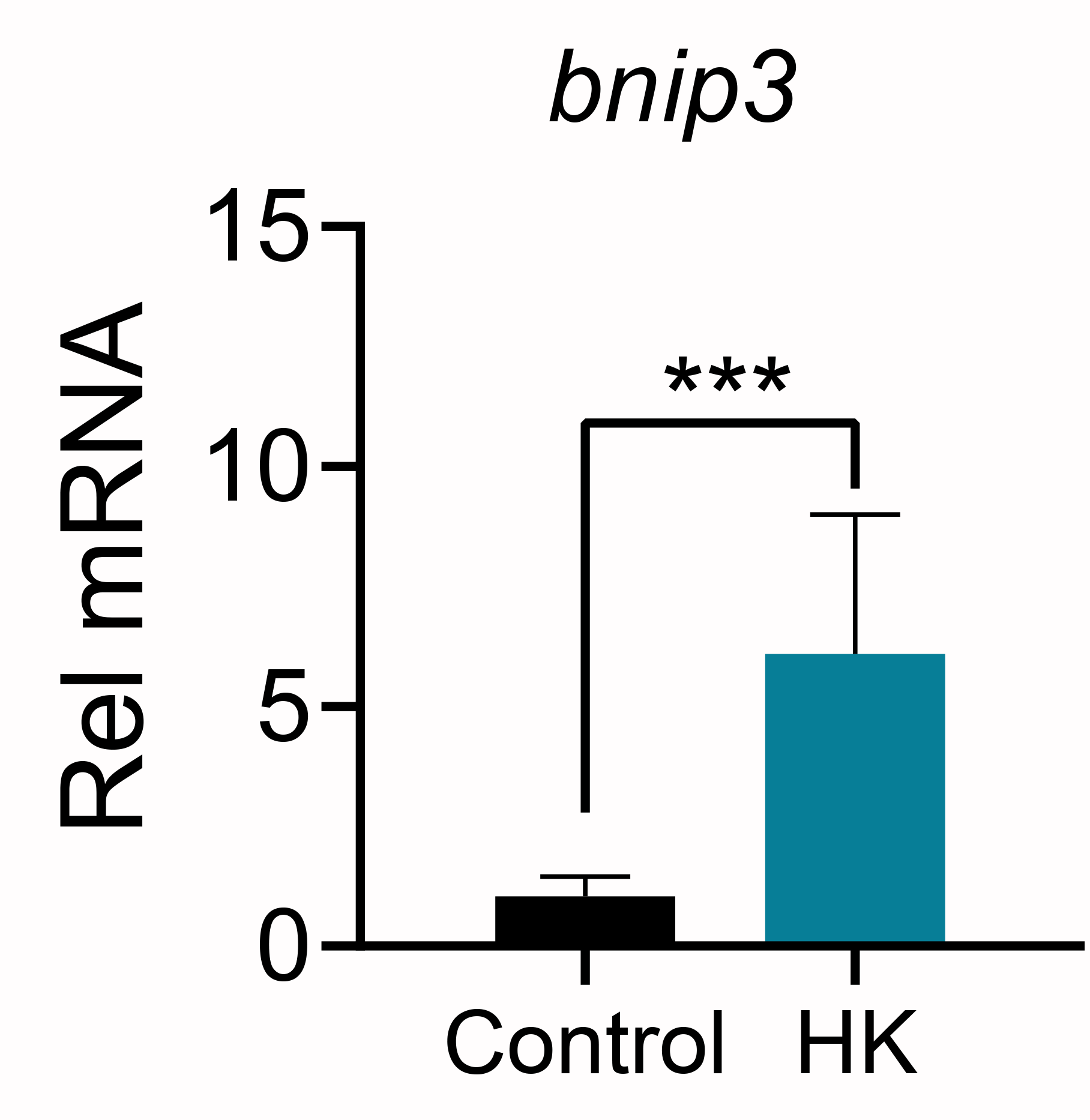

Supplement: Supplementary file 1 [file bioengineering-12-00434-s001.zip › Figure S9 bnip3 express validation.tif]
